# Short-term subjective and EEG responses following cervical rotatory manipulation are not reproduced by simulated cracking sound: A randomized controlled study

**DOI:** 10.1371/journal.pone.0352526

**Published:** 2026-10-14

**Authors:** Yuanxun Lin, Weixing Zhong, Meiling Yu, Yaoshuai Yu, Lixin Tang, Fan Xue, Jundan Wei, Junhua Li, Yikai Li

**Affiliations:** 1 School of Traditional Chinese Medicine, Southern Medical University, Guangzhou, Guangdong, China; 2 Department of Traditional Chinese Medicine, The Fifth Affiliated Hospital of Southern Medical University, Guangzhou, Guangdong, China; 3 Department of Traditional Chinese Medicine, Shanghai Municipal Hospital of Traditional Chinese Medicine, Shanghai, China; 4 Department of Orthopedic Surgery, The Affiliated TCM Hospital of Guangzhou Medical University, Guangzhou, Guangdong, China; IMU University, MALAYSIA

## Abstract

**Background:**

Cervical rotatory manipulation (CRM) is commonly used for neck-related symptoms and is often accompanied by a cracking sound. Whether a simulated cracking sound can reproduce the short-term subjective and neurophysiological responses to the complete CRM remains unclear.

**Objective:**

This study compared short-term subjective and electroencephalography (EEG) responses to CRM with those following sham manipulation with or without a simulated cracking sound.

**Methods:**

In this randomized controlled trial with three parallel groups and a single intervention session, 54 healthy volunteers were allocated to CRM, sham manipulation, or sham + simulated sound. Positive affect, negative affect, comfort, satisfaction, and eyes closed resting state EEG were assessed before and after the intervention. Prespecified EEG derived outcomes included frontal alpha power, frontal alpha/beta ratio, occipital individual alpha frequency, and alpha band frontoparietal and frontotemporal functional connectivity.

**Results:**

CRM produced greater improvements in positive affect, comfort, and satisfaction than sham manipulation or sham + simulated cracking sound, whereas no clear difference among groups was observed for negative affect. Differences among groups were also observed in frontal alpha/beta ratio and alpha band frontoparietal and frontotemporal functional connectivity. Sham + simulated sound did not reproduce the overall pattern of subjective and EEG responses observed after CRM.

**Conclusions:**

CRM produced greater improvements over the short-term in positive affect, comfort, and satisfaction and was accompanied by differences in selected resting state EEG outcomes compared with sham manipulation with or without simulated cracking sound. Adding simulated cracking sound to sham manipulation did not reproduce this pattern. Because CRM differed from the control conditions in several procedural components, the specific component or combination of components responsible for these differences could not be determined.

**Trial registration:**

The trial was registered with the International Traditional Medicine Clinical Trial Registry (URL: https://itmctr.ccebtcm.org.cn; ITMCTR2025001112).

## Introduction

Cervical rotatory manipulation (CRM) is a commonly used technique in traditional Chinese orthopedic manipulation for adjusting the functional state of the cervical facet joints [1]. Clinically, it is frequently applied for nonspecific neck pain [2], neck and shoulder discomfort [3], dizziness [4], and other cervical spondylosis-related symptoms [5]. From a biomechanical perspective, CRM shares characteristics with high velocity, low amplitude manipulation, in which a rapid, small amplitude thrust is delivered after the targeted cervical segment reaches the elastic barrier, followed by immediate release [6]. In clinical practice, patients often report a short-term sense of “release” or relaxation after CRM, accompanied by reduced tension, increased comfort, and improved bodily experience. These short-term subjective changes are commonly considered an important component of the therapeutic effects of CRM [7]. However, the neurophysiological changes accompanying the subjective responses observed shortly after CRM remain unclear. CRM is not a single stimulus but a complex sensory event involving contact between the therapist and participant, cervical segment positioning, cutaneous and soft tissue somatosensory stimulation, cervical proprioceptive input, patient expectancy, and, in some cases, joint cavitation during the manipulation, which is commonly perceived as a cracking sound [8]. Patients and even some therapists often regard this sound as a sign of successful manipulation [9]. Nevertheless, whether the cracking sound itself makes a substantial contribution to these subjective responses, or merely acts as a contextual cue that enhances patients’ expectations and treatment credibility, remains uncertain. Relying solely on subjective feedback after CRM may therefore make it difficult to distinguish responses to the complete manipulation procedure from responses associated with the cracking sound, expectancy, or other contextual factors [10]. A controlled experimental design is therefore needed to determine whether adding a simulated cracking sound to sham manipulation can reproduce the subjective and neurophysiological responses observed after the complete CRM procedure.

Electroencephalography recorded at rest provides an objective approach for characterizing changes in spontaneous neural activity following an intervention [11]. In the present study, we focused on frontal alpha power, the frontal alpha/beta ratio, and occipital individual alpha frequency (IAF). Frontal alpha activity during rest with the eyes closed has been examined in relation to cortical arousal, internally directed attention, and sensory processing [12]. The frontal alpha/beta ratio has been examined as a relative measure of spectral activity associated with differences in arousal and attentional state [13,14]. Occipital IAF reflects the peak frequency of the dominant individual alpha rhythm and is an important indicator of posterior dominant alpha activity during eyes closed rest [15]. Unlike measures based on spectral power, IAF primarily reflects the individualized frequency organization of alpha rhythms and resting-state brain functional characteristics, and provides information about changes in alpha rhythm organization that is complementary to changes in spectral power. In addition to spectral measures, we included alpha-band fronto-parietal and fronto-temporal weighted phase lag index (wPLI) as functional connectivity measures [16]. wPLI reduces the influence of volume conduction on phase synchrony estimates and provides a measure of functional coupling between brain regions [17]. Together, frontal alpha power, the frontal alpha/beta ratio, occipital IAF, and alpha band wPLI connectivity provide complementary information on spectral activity, relative frequency band activity, individual alpha rhythm characteristics, and interregional phase synchronization.

Previous studies on CRM or related cervical manipulation techniques have mainly focused on clinical analgesia, improvement in joint range of motion, or outcomes reported by patients [1,2,4,18]. Although these outcomes are clinically important, they do not directly clarify whether a single manipulation session can induce measurable changes in resting state EEG activity. However, few studies have directly examined whether a simulated cracking sound can reproduce responses observed after cervical manipulation [10,19]. This distinction is important because a simulated cracking sound reproduces the auditory cue but does not reproduce the complete CRM procedure, which includes several additional procedural components. Therefore, determining whether the simulated cracking sound reproduces the subjective and EEG responses observed after the complete CRM procedure may clarify the contribution of the sound cue without assuming a specific mechanism [20].

To address this question, we conducted a single-session, three-arm, parallel randomized controlled EEG study in healthy volunteers. Participants were randomly assigned to CRM, sham manipulation, or sham + simulated sound. We hypothesized that the complete CRM procedure would produce greater short-term changes in positive affect, comfort, satisfaction with the procedure, and the prespecified resting state EEG metrics than either control condition. The primary aim was to determine whether adding a simulated cracking sound to sham manipulation could reproduce the subjective and EEG responses observed following the complete CRM procedure. The study was not designed to isolate the independent contributions of soft tissue release, positioning at end range, traction, HVLA thrust, proprioceptive stimulation, vestibular input, or naturally occurring cavitation.

## Materials and methods

### Study design

This study was designed as a randomized controlled trial with three parallel groups and a single intervention session. Eligible participants were randomly allocated in a 1:1:1 ratio to the CRM group, sham group, or sham + simulated sound group. Participants were blinded to the study hypothesis. Outcome assessors, personnel responsible for EEG preprocessing, and data analysts were blinded to group allocation. However, full participant blinding to the presence or absence of an HVLA thrust was not feasible because the rapid thrust constituted a physically perceptible component of the CRM procedure. The study was reported in accordance with the CONSORT statement. The participant flow diagram is shown in Fig 1, and the CONSORT checklist is provided in S1 File.

**Fig 1:**
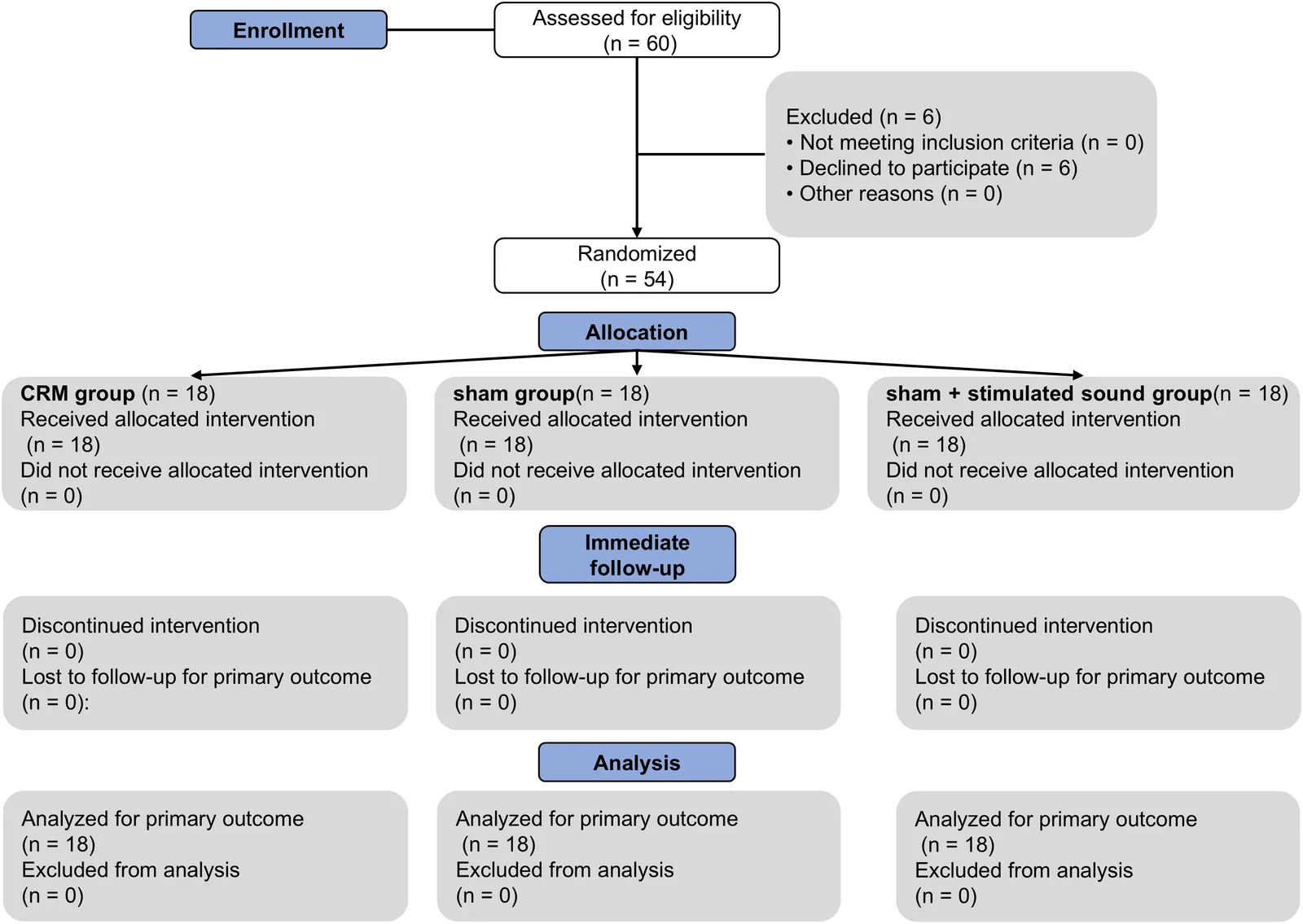
CONSORT flow diagram of participant enrollment, allocation, follow-up, and analysis.

### Participants

From April to August 2025, 60 healthy volunteers were recruited through advertisements posted in the Orthopedics and Traumatology outpatient department of The First Affiliated Hospital of Guangzhou Medical University. The study protocol was approved by the Ethics Committee of The Affiliated TCM Hospital of Guangzhou Medical University (Approval No. 2025NK022; 7 March 2025). The trial was prospectively registered with the International Traditional Medicine Clinical Trial Registry (Registration No. ITMCTR2025001112; 23 March 2025). All participants provided written informed consent before enrollment.

The inclusion criteria were as follows: (1) age between 20 and 50 years; (2) no cervical disc herniation or other cervical structural lesions requiring treatment, as confirmed by imaging and clinical screening; (3) no history of tumor, major trauma, or cervical spine surgery; (4) body mass index (BMI) < 26 kg/m^2^; (5) Self-Rating Anxiety Scale (SAS) standard score < 53 and Self-Rating Depression Scale (SDS) standard score < 53; (6) no massage, manipulation, physical therapy, or related interventions within the previous month; (7) willingness to participate and ability to provide written informed consent.

The exclusion criteria were as follows: (1) imaging evidence of cervical deformity or high-risk anatomical abnormalities, such as block vertebrae, os odontoideum, or basilar invagination; (2) severe cardiovascular or cerebrovascular disease, coagulation disorders, or other conditions that might increase the risk of cervical manipulation; (3) history of cranial or spinal trauma or surgery; (4) autoimmune or infectious diseases; (5) pregnancy or lactation; (6) implanted electronic devices, metal implants, or other conditions that might affect EEG acquisition or intervention safety; (7) marked fear of manipulation or inability to cooperate with eyes-closed resting state EEG recording.

Participants could withdraw consent at any time. Safety was monitored during and immediately after the intervention through direct observation and active inquiry. Prespecified adverse events included neck pain or discomfort, dizziness, nausea, upper limb numbness, transient neurological symptoms, and skin discomfort. The onset, duration, severity, management, outcome, and possible relation to the intervention were documented.

### Randomization, allocation concealment, and blinding

An independent researcher generated the randomization sequence using a computer based random number generator with randomly permuted block sizes of 6 and 9 and an allocation ratio of 1:1:1. Allocation was concealed using sequentially numbered, opaque, sealed envelopes prepared by personnel who were not involved in recruitment, intervention delivery, outcome assessment, EEG preprocessing, or statistical analysis. After completion of baseline assessments, the therapist opened the next envelope in numerical order. EEG datasets were coded using participant and time point identifiers without group labels. Group allocation remained concealed from personnel performing EEG preprocessing and statistical analyses until the preprocessing outputs had been finalized. All participants wore the same Newman M110 bone conduction headset during the intervention. No sound was played in the CRM or sham groups. Participants in the sham + simulated sound group received a computer controlled simulated cracking sound. At study completion, participants indicated whether they believed that they had received CRM by selecting “yes,” “unsure,” or “no.” This single nonvalidated question was treated as an exploratory measure of treatment belief rather than formal evidence of successful blinding or treatment credibility.

### Intervention

All interventions were delivered in a single session by a researcher with more than 5 years of clinical experience in CRM. Before the principal intervention procedure, all participants received approximately 15 s of therapist contact. In the CRM group, this consisted of active soft tissue release as part of the complete CRM procedure, whereas participants in the two control groups received light contact of similar duration without therapeutic soft tissue techniques. The active soft tissue component was not reproduced in the control groups because the study was designed to compare the complete CRM procedure with sham conditions rather than to isolate the effect of the HVLA thrust.

#### CRM group.

Participants were seated, and the operator stood behind them. Before the thrust procedure, a brief standardized soft tissue release was applied for approximately 15 s. This release mainly consisted of acupoint-based pressing, kneading, and plucking techniques. The operator used the thumb to press and knead the paraspinal muscles around the cervical spinous processes. When tender points or palpable cord-like taut bands were encountered, the thumb was used to gently pluck the surrounding soft tissue. After the soft tissue release, participants were instructed to flex the neck forward. The operator placed the thumb of one hand against one side of the C5 - C6 level, with the remaining fingers positioned on the contralateral side, while the other hand supported the participant’s mandible. Because the participants were healthy volunteers without a clinically indicated symptomatic side, CRM was performed bilaterally, with the same standardized procedural sequence applied to both the left and right sides. For each side, the operator applied slow upward traction and rotated the head approximately 45°. After the elastic barrier was reached, the operator briefly paused and delivered a controlled, rapid, small-amplitude thrust with an additional rotation of approximately 5°. All participants completed the planned bilateral procedure, and no deviations from the standardized intervention were recorded. This procedure could be accompanied by a cracking sound. No sound was played through the bone-conduction headset in the CRM group. No repeated thrust was performed solely to elicit an audible cracking sound. However, the occurrence, number, timing, and acoustic characteristics of naturally occurring audible cavitation events during CRM were not prospectively recorded or standardized. Accordingly, the CRM condition represented the complete clinical procedure rather than an isolated HVLA thrust.

#### Sham group.

The sham manipulation procedure was designed to match the CRM condition as closely as possible in participant positioning, therapist contact, contact points, procedural duration, and standardized verbal instructions. Participants were seated in the same position, and the operator stood behind them. The same bone-conduction headset was worn, but no sound was played. Before passive neck positioning, a matched 15 s light manual contact procedure was applied around the cervical paraspinal region. This procedure involved gentle contact only, without deep pressing, kneading, plucking of taut bands, or therapeutic soft tissue release. The operator then placed the hands in positions similar to those used in the CRM group and gently positioned the participant’s neck within a comfortable range. However, the procedure did not approach the restrictive barrier, and no HVLA thrust was delivered. Passive positioning and gentle stretching were applied bilaterally within a comfortable range, without approaching the elastic barrier or delivering an HVLA thrust. All participants completed the planned sham procedure, and no procedural deviations were recorded.

#### Sham + simulated sound group.

The sham manipulation procedure was identical to that used in the sham group, including the matched 15-s light manual contact procedure before passive neck positioning. During passive neck rotation, a brief standardized bone-conducted sound burst was delivered through the Newman M110 headset to mimic a cracking sound. Each burst lasted approximately 0.2–0.5 s and was delivered 1–2 times per session, synchronized with the peak rotation moment. The waveform and playback intensity were computer-controlled and kept constant across participants. Playback intensity was standardized through pretrial technical calibration to ensure audibility and comfort. This was not a formal validation study, and perceived realism, intensity, anatomical location, and similarity to naturally occurring cavitation were not systematically rated. No HVLA thrust was delivered in this group.

### Assessment procedure and subjective outcomes

Participants first completed a 15 min acclimatization period with their eyes closed. Baseline EEG was then recorded for 3 min and defined as P1. The EEG cap was removed after P1 in all participants because the interventions required access to the occipital and cervical regions. After the assigned intervention, the cap was reapplied using the same electrode configuration and anatomical positioning procedure. Reapplication required approximately 3 min, and P2 EEG recording began approximately 4 min after completion of the intervention and continued for 3 min. The EEG assessment therefore represented a period approximately 4–7 min after the intervention.

Subjective outcomes included the Adapted International Positive and Negative Affect Schedule Short Form (Adapted I-PANAS-SF), an eight item State Comfort Scale, and a ten item Procedure Related Satisfaction Scale developed by the investigators [21–23]. The Adapted I PANAS SF was administered in Chinese through a mini program using a continuous slider ranging from 1 to 10. Five positive affect items and five negative affect items were summed separately, yielding a theoretical range of 5–50 for each domain. Recorded values, including decimal values, were analyzed without rounding, recategorization, or conversion. The instrument basis, Chinese wording, response format, scoring procedure, psychometric considerations, and study specific internal consistency are provided in S2 File. The State Comfort Scale had a total score from 0 to 32, and the Procedure Related Satisfaction Scale had a total score from 0 to 100, with higher scores indicating greater comfort or satisfaction. At P1, satisfaction referred to the experimental procedures and bodily experience encountered before the assigned intervention, whereas the P2 assessment additionally included the intervention. The P2 minus P1 difference therefore represented a change in cumulative procedure related satisfaction rather than a pure measure of treatment satisfaction or relaxation. Both scales were considered exploratory measures developed by the investigators. Their complete content, scoring, development, and psychometric limitations are provided in S3 File.

### EEG acquisition and preprocessing

EEG was recorded using a 32 channel EMOTIV EPOC Flex system (Emotiv, California, USA) at a sampling rate of 128 Hz, with electrode positions based on the international 10–20 system. Scalp channels were recorded relative to the CMS electrode, with the DRL electrode used for common mode noise cancellation. After artifact removal and interpolation of bad channels, recordings were rereferenced to the average reference. Before both P1 and P2 recordings, electrode contact was adjusted using conductive gel until all 32 recording channels displayed green, indicating good contact, on the Emotiv PRO contact quality map. The device does not provide numerical impedance values; therefore, quantitative impedance comparisons between P1 and P2 were unavailable. Following cap reapplication, the same electrode configuration and anatomical positioning procedure were used and the full contact quality check was repeated before P2.

EEG preprocessing was performed in MATLAB 2013b using EEGLAB [24]. Continuous data were filtered from 0.1 to 30 Hz. No separate notch filter was applied because the 30 Hz low pass cutoff excluded 50 Hz mains activity. After removal of the first and last 5 s of each recording, data were divided into nonoverlapping 2 s epochs. Bad channel screening, automated and visual artifact rejection, independent component analysis, ADJUST assisted component identification, interpolation, and average rereferencing were performed using the same predefined criteria for all participants and time points. Recordings with at least 120 s of artifact free data were retained. All 108 EEG recordings met the predefined quality criterion, and no participant was excluded because of EEG quality. Retained data duration and epoch rejection rates were comparable across groups and time points. Complete acquisition parameters, preprocessing thresholds, predefined EEG regions of interest, feature extraction settings, and quality control summaries are provided in S4 File. The complete stepwise preprocessing pipeline and relevant EEGLAB functions are provided in S5 File.

### EEG outcomes

The 5 prespecified EEG outcomes were frontal alpha power, frontal alpha/beta ratio, occipital IAF, alpha band frontoparietal wPLI, and alpha band frontotemporal wPLI. Predefined electrode compositions for the frontal, parietal, temporal, and occipital regions are reported in S4 File. Power spectral density was estimated from retained EEG epochs using Welch’s method with a Hamming window. Frontal alpha power was calculated over 8–13 Hz, frontal beta power over 13–30 Hz, and the frontal alpha/beta ratio was calculated at each time point. The primary spectral power analysis used the untransformed absolute power scale without relative power normalization. Occipital IAF was defined as the frequency of the maximum power spectral density peak within 8–13 Hz.

For connectivity analyses, preprocessed EEG was filtered to 8–13 Hz and analytic signals were obtained using the Hilbert transform. wPLI was calculated for individual channel pairs, averaged across retained segments, and subsequently averaged across predefined frontal to parietal and frontal to temporal region pairs [25]. Whole matrix connectivity results were used for exploratory visualization only. Change scores for all prespecified EEG outcomes were calculated as Δ = P2 - P1. Detailed feature definitions and computational parameters are provided in S4 File. The primary subjective outcome was the P1 to P2 change in positive affect assessed using the Adapted I PANAS SF. Secondary subjective outcomes included changes in negative affect, comfort, and procedure related satisfaction. EEG outcomes were treated as prespecified neurophysiological outcomes rather than specific markers of relaxation.

### Sample size estimation

An a priori sample size calculation was performed using G*Power version 3.1. The test family was specified as *F* tests and the statistical test as fixed effects omnibus one way analysis of variance, corresponding to the omnibus comparison of changes in positive affect among the three groups. Because G*Power does not directly implement the linear mixed effects model used for the final primary analysis, analysis of variance of P2 minus P1 change scores was used as an approximation for sample size planning. Based on preliminary work, an omnibus standardized effect size of Cohen’s *f* = 0.55 was assumed. With three groups, α = 0.05, 90% power, and equal allocation, the required sample size was 45 participants, or 15 per group. 60 volunteers were assessed for eligibility, 6 declined participation before randomization, and 54 participants were randomized.

### Statistical analysis

All analyses were performed using GraphPad Prism 9.0 and R version 4.3.1. Continuous variables are presented as means ± standard deviation (SD), and categorical variables as counts and percentages. Baseline characteristics were compared using one way analysis of variance, Pearson’s chi square test, or Fisher’s exact test, as appropriate. Change scores were calculated as Δ = P2 − P1. Treatment belief responses were compared among groups using an extension of Fisher’s exact test for the 3 × 3 contingency table because several expected cell counts were below five. This post hoc analysis was considered exploratory, and a nonsignificant result was not interpreted as evidence of successful blinding. Subjective outcomes were analyzed using linear mixed effects models with group, time, and group × time interaction as fixed effects and participant ID as a random intercept. The interaction was the primary test of differential change among groups. Significant interactions were followed by prespecified pairwise comparisons. When the interaction was not significant, the full model was retained and main effects were not interpreted as differential intervention effects. The five prespecified EEG change scores were analyzed separately using one way analysis of variance with intervention group as the between participant factor. Subjective outcomes were analyzed at the original P1 and P2 levels to account for repeated observations, whereas EEG outcomes were analyzed as participant level change scores. Model assumptions were assessed by visual inspection of residual distributions and residual versus fitted value plots. Model convergence was also checked for the linear mixed effects models. Homogeneity of variance for the one way analyses of variance was evaluated using Levene’s test, and influential observations were examined using standardized residuals and Cook’s distance where applicable. Prespecified contrasts were calculated as sham minus CRM, sham + simulated sound minus CRM, and sham + simulated sound minus sham. Effect estimates are reported with 95% confidence interval (CI). EEG pairwise effect sizes are reported as Cohen’s d using the pooled SD of change scores. Pairwise *P* values were unadjusted unless otherwise specified. Brain behavior associations between changes in positive affect and changes in the five EEG outcomes were examined using partial Spearman correlations accounting for intervention group and sex. Variables were rank transformed, group and sex effects were removed using linear models, and correlations were calculated from the residuals. Ninety five percent CI were estimated using 5,000 bootstrap resamples stratified by group and sex. As an exploratory sensitivity analysis, each EEG change score was additionally analyzed using a linear model including intervention group and sex as main effects. Group × sex interactions were not included. Overall group effects were evaluated using Type II *F* tests, and sex adjusted estimated marginal means and pairwise differences are reported with 95% CI. The Benjamini and Hochberg false discovery rate procedure was applied separately to three families of five tests: the primary EEG omnibus group effects, the partial Spearman correlations, and the exploratory sex adjusted EEG group effects. Subjective outcome interactions, pairwise comparisons, equivalence analyses, and the blinding assessment were not included in these families. Exploratory equivalence analyses compared sham + simulated sound with sham using the two one sided tests procedure. Because established minimally important differences were unavailable, the smallest effect size of interest was defined as Cohen’s *d* = ± 0.20 and converted to the original units using the pooled SD of change scores. Equivalence was concluded only when the 90% CI for the between group difference fell entirely within the corresponding margins. These analyses were considered exploratory because the margins were based on observed variability rather than externally validated thresholds. CRM and P1 were used as the reference categories for intervention group and time, respectively. Participant sex was included as a categorical main-effect covariate in the exploratory sex-adjusted analyses. Full model specifications are provided in S11 File.

## Results

### Participant flow and baseline characteristics

A total of 60 healthy volunteers were assessed for eligibility. Six participants declined to participate, and 54 participants were randomized equally to the CRM group, sham group, or sham + simulated sound group, with 18 participants in each group. All randomized participants completed the P1 and P2 assessments and were included in the analyses. No subjective or EEG derived outcome data were missing, and no imputation was required. No adverse events were observed in any of the three intervention groups during or immediately after the intervention. Baseline demographic characteristics were comparable among the three groups. No significant between group differences were observed in age, weight, height, BMI, or sex distribution (all *P* > 0.05; Table 1). The CRM, sham, and sham + simulated sound groups included 8, 9, and 8 male participants and 10, 9, and 10 female participants, respectively. Sex distribution did not differ significantly among the groups (*P* = 0.928).

**Table 1:** Baseline demographic characteristics of participants.

Variable	CRM (n = 18)	sham (n = 18)	sham + simulated sound (n = 18)	*P* value
**Age (years)**	24.17 ± 3.54	23.70 ± 3.40	23.81 ± 2.24	0.548
**Weight (kg)**	60.16 ± 13.86	63.01 ± 15.71	62.11 ± 15.40	0.341
**Height (m)**	1.67 ± 0.08	1.68 ± 0.08	1.67 ± 0.07	0.262
**BMI (kg/m**^**2**^)	23.93 ± 3.93	23.49 ± 4.01	23.85 ± 3.76	0.584
**Sex, n (%)**				
**Male**	8(44.44)	9(50)	8(44.44)	
**Female**	10(55.56)	9(50)	10(55.56)	0.928

Continuous variables are presented as mean ± SD, and sex is presented as n (%). *P* values were obtained using one-way analysis of variance for continuous variables and Pearson’s chi-square test for sex.

CRM, cervical rotatory manipulation; BMI, body mass index.

*Significant effect (*P* < 0.05).

### Blinding assessment

Treatment belief responses are presented in Table 2 and Fig 2A. 14 participants in the CRM group (77.8%), 10 in the sham group (55.6%), and 11 in the sham + simulated sound group (61.1%) believed that they had received CRM. The response distributions did not differ significantly among groups according to an extension of Fisher’s exact test for the 3 × 3 contingency table (*P* = 0.241). However, the modest sample size and use of a single nonvalidated question preclude interpreting this result as evidence of successful participant blinding.

**Table 2:** Participants’ beliefs regarding whether they received CRM.

Group	CRM (n = 18)	sham (n = 18)	sham + simulated sound (n = 18)	*P* value
**Believed they received CRM**	14 (77.8%)	10 (55.6%)	11 (61.1%)	
**Unsure whether they received CRM**	3 (16.7%)	6 (33.3%)	2 (11.1%)	
**Did not believe they received CRM**	1 (5.6%)	2 (11.1%)	5 (27.8%)	0.241

Data are presented as n (%), with percentages calculated within each intervention group. The *P* value was obtained using an extension of Fisher’s exact test for the 3 × 3 contingency table. This exploratory analysis assessed treatment belief rather than formally establishing successful participant blinding.

CRM, cervical rotatory manipulation.

*Significant effect (*P* < 0.05).

**Fig 2:**
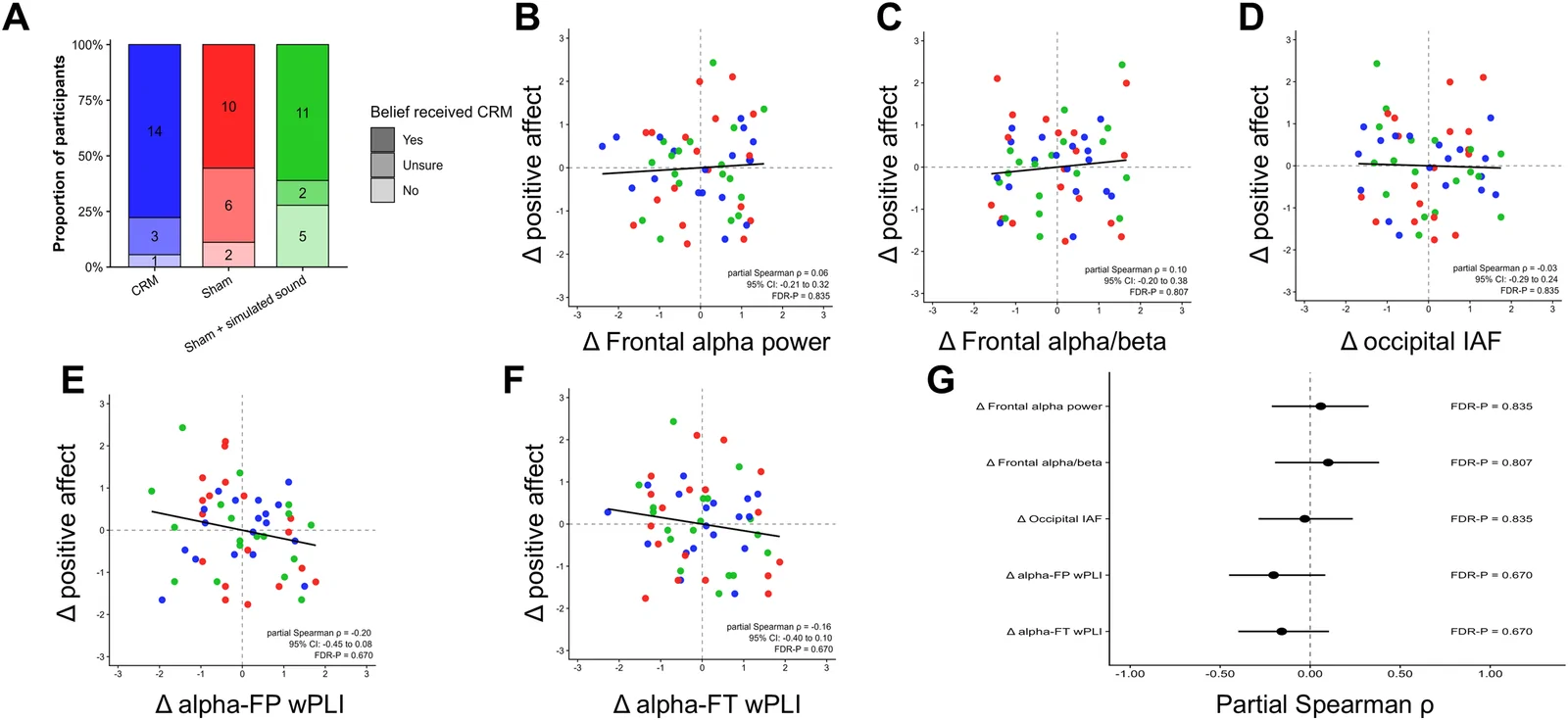
Study procedure and primary subjective and EEG outcomes. (A) Schematic illustration of the experimental procedure. Participants were randomized to CRM, sham manipulation, or sham manipulation +simulated sound. Eyes-closed resting-state EEG was recorded before intervention (P1) and after intervention (P2). Because the EEG device was removed and reapplied during the intervention, post-intervention EEG acquisition began approximately 4 min after completion of the intervention and continued for 3 min, corresponding to approximately 4–7 min after the intervention. (B-E) Subjective outcomes at P1 and P2, including positive affect, negative affect, comfort, and satisfaction. (F-I) Changes in prespecified EEG-derived outcomes, including frontal alpha power, frontal alpha/beta ratio, alpha-band fronto-parietal wPLI, and alpha-band fronto-temporal wPLI. (J) Points and horizontal lines represent the estimated differences in change scores and their 90% confidence intervals for positive affect, comfort, and satisfaction between the sham + simulated sound group and the sham group. The vertical solid line at 0 indicates no between-group difference. The shaded regions indicate the operationally defined SESOI bounds. Values to the left of 0 favor the sham group, whereas values to the right of 0 favor the sham + simulated sound group. CRM, cervical rotatory manipulation; EEG, electroencephalography; wPLI, weighted phase lag index; FP, fronto-parietal; FT, fronto-temporal; CI, confidence interval. ns, not significant; *P < 0.05; **P < 0.01; ***P < 0.001; ****P < 0.0001.

### Primary and secondary subjective outcomes

A significant group × time interaction was observed for positive affect. Positive affect increased markedly in the CRM group, whereas only a small increase was observed in the sham group and a slight decrease was observed in the sham + simulated sound group (Table 3; Fig 3B). Pairwise comparisons showed that the increase was greater in the CRM group than in both control groups. The increase in positive affect was also smaller in the sham + simulated sound group than in the sham group. For negative affect, the group × time interaction was not statistically significant, providing no evidence that the pre-to-post change differed among the three groups (Table 3; Fig 3C). Comfort increased in all three groups, with a significant group × time interaction and a greater increase in the CRM group than in both control groups (Table 3; Fig 3D). Satisfaction also showed a significant group × time interaction, with the greatest increase observed in the CRM group (Table 3; Fig 3E). Detailed P1 and P2 descriptive statistics and pairwise between-group comparisons of change scores are provided in S9 File.

**Table 3:** Changes in subjective outcomes across the three intervention groups.

Outcome	CRM Δ(mean ± SD)	sham Δ(mean ± SD)	sham + simulated sound Δ(mean ± SD)	Group × time *F*	*P* value
Adapted I-PANAS-SF (positive affect)	8.06 ± 3.24	1.10 ± 3.42	−1.15 ± 2.45	44.12	<0.001*
Adapted I-PANAS-SF (negative affect)	−0.09 ± 1.69	−0.65 ± 1.75	−0.50 ± 1.17	0.64	0.530
Comfort	4.12 ± 4.33	1.50 ± 2.72	1.54 ± 2.34	4.15	0.019*
Satisfaction	11.39 ± 7.64	2.29 ± 6.42	−1.48 ± 7.62	16.01	<0.001*

Data are presented as mean ± SD. Change scores were calculated as Δ = P2 − P1. Group × time effects were tested using linear mixed-effects models. The *P* values reported in this table correspond to the omnibus group × time interaction tests and are unadjusted. Detailed P1 and P2 descriptive statistics and pairwise between-group comparisons of change scores, including mean differences and 95% confidence intervals, are provided in S9 File.

CRM, cervical rotatory manipulation; Adapted I-PANAS-SF, Adapted International Positive and Negative Affect Schedule-Short Form.

*Significant effect (*P* < 0.05).

**Fig 3:**
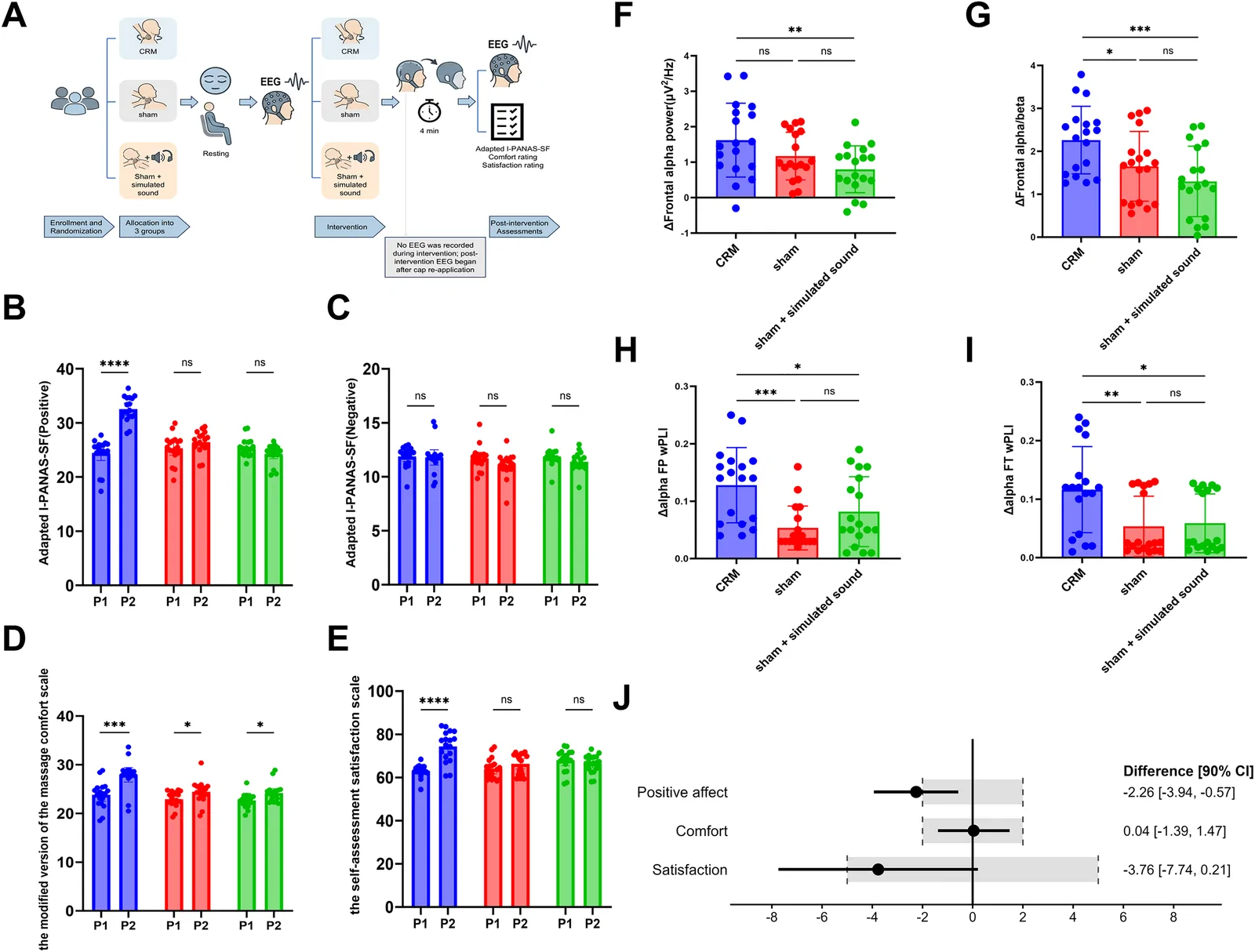
Blinding assessment and exploratory brain–behavior association analyses. (A) Participants’ beliefs regarding whether they had received CRM after the intervention. (B–F) Partial Spearman residual plots showing associations between changes in positive affect and changes in frontal alpha power, frontal alpha/beta ratio, occipital IAF, alpha-band frontoparietal wPLI, and alpha-band frontotemporal wPLI after adjustment for intervention group and participant sex. The continuous variables were rank-transformed, and the plotted values represent standardized residuals obtained after removing the effects of intervention group and sex. Each point represents one participant, and colors indicate intervention groups. Black lines represent fitted linear trends between the adjusted rank residuals. Partial Spearman coefficients and 95% confidence intervals estimated using 5,000 bootstrap resamples stratified by intervention group and sex are shown in each panel. (G) Forest plot summarizing the partial Spearman coefficients, bootstrap 95% confidence intervals, and Benjamini–Hochberg FDR-adjusted *P* values for the five prespecified associations. Benjamini–Hochberg FDR correction was applied across the five partial Spearman association tests shown in panels B–F. The FDR-adjusted *P* values are displayed in the individual panels and summarized in panel G. CRM, cervical rotatory manipulation; IAF, individual alpha frequency; wPLI, weighted phase lag index; FP, fronto-parietal; FT, fronto-temporal; FDR, false discovery rate.

### EEG-derived outcomes

Frontal alpha power differed significantly among the three groups, with a greater increase in the CRM group than in the sham + simulated sound group; the difference between the CRM and sham groups was not statistically significant (Table 4; Fig 3F). The frontal alpha/beta ratio also showed a significant group effect. The increase was greater in the CRM group than in both the sham and sham + simulated sound groups, whereas the two control groups did not differ significantly (Table 4; Fig 3G). Occipital IAF did not differ significantly among the three groups (Table 4). Significant group differences were observed for alpha-band frontoparietal wPLI, with greater increases in the CRM group than in both control groups (Table 4; Fig 3H). A similar pattern was observed for alpha-band frontotemporal wPLI (Table 4; Fig 3I). In the exploratory sex-adjusted sensitivity analyses, the overall group effects remained significant for frontal alpha power, frontal alpha/beta ratio, alpha-FP wPLI, and alpha-FT wPLI after FDR correction, whereas occipital IAF remained nonsignificant. Detailed sex-adjusted estimates and confidence intervals are provided in S8 File, and detailed unadjusted pairwise comparisons and effect sizes from the primary EEG analyses are provided in S10 File. Exploratory whole-scalp visualizations of alpha-band power changes are shown for the CRM, sham, and sham + simulated sound groups in Fig 4A-C, respectively.

**Table 4:** Summary of changes and omnibus group comparisons for prespecified EEG-derived outcomes.

EEG-derived outcome	CRM Δ(mean ± SD)	sham Δ(mean ± SD)	sham + simulated sound Δ(mean ± SD)	Overall *P*	FDR-adjusted *P*
Frontal alpha power (μV^2^/Hz)	1.62 ± 1.04	1.17 ± 0.67	0.80 ± 0.66	0.014	0.018
Frontal alpha/beta ratio	2.26 ± 0.79	1.65 ± 0.81	1.30 ± 0.82	0.003	0.007
Occipital IAF (Hz)	0.37 ± 0.21	0.40 ± 0.15	0.37 ± 0.18	0.855	0.855
Alpha-FP wPLI	0.13 ± 0.07	0.05 ± 0.04	0.08 ± 0.06	<0.001	0.005
Alpha-FT wPLI	0.12 ± 0.08	0.05 ± 0.05	0.06 ± 0.05	0.004	0.007

Data are presented as mean ± SD. Change scores were calculated as Δ = P2 - P1. Each EEG-derived change score was analyzed using a separate one-way analysis of variance with intervention group as the between-participant factor. Benjamini–Hochberg false discovery rate correction was applied across the five omnibus group-effect tests. Detailed pairwise comparisons, mean differences with 95% confidence intervals, unadjusted *P* values, and Cohen’s *d* effect sizes are provided in S10 File.

EEG, electroencephalography; IAF, individual alpha frequency; wPLI, weighted phase lag index; FP, fronto-parietal; FT, fronto-temporal; CRM, cervical rotatory manipulation.

*Significant effect (*P* < 0.05).

**Fig 4:**
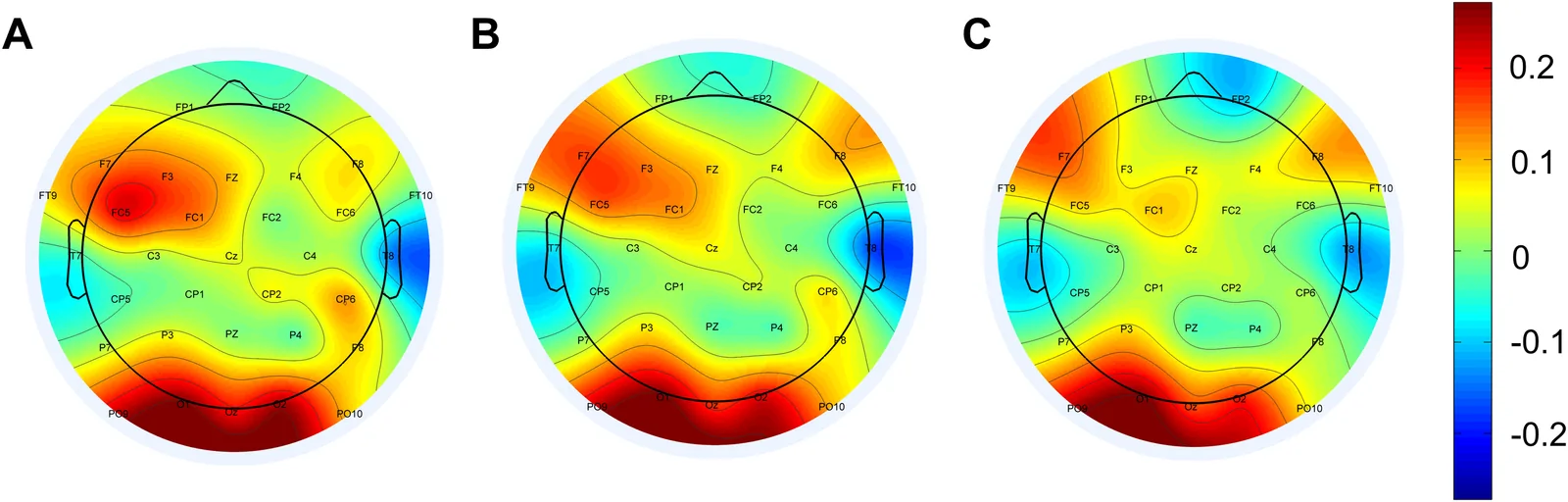
Scalp topography of alpha-band power changes after intervention. Scalp topographic maps show group-level alpha-band power changes from P1 to P2 (Δ = P2 - P1). (A) CRM group. (B) sham group. (C) sham + simulated sound group. Warm colors indicate relative increases in alpha power, whereas cool colors indicate relative decreases. The same color scale was applied across the three groups to facilitate visual comparison. The frontal region was used as the prespecified region of interest for statistical analysis. CRM, cervical rotatory manipulation.

### Exploratory brain-behavior associations

Partial Spearman analyses were performed to examine participant-level associations between changes in positive affect and changes in the five prespecified EEG-derived outcomes after accounting for intervention group and participant sex. None of the five associations remained statistically significant after FDR correction. The partial Spearman coefficient was ρ = 0.06 for frontal alpha power (95% CI −0.21 to 0.32; FDR-adjusted *P* = 0.835; Fig 2B), ρ = 0.10 for the frontal alpha/beta ratio (95% CI −0.20 to 0.38; FDR-adjusted *P* = 0.807; Fig 2C), and ρ = −0.03 for occipital IAF (95% CI −0.29 to 0.24; FDR-adjusted *P* = 0.835; Fig 2D). The corresponding coefficients were ρ = −0.20 for alpha-FP wPLI (95% CI −0.45 to 0.08; FDR-adjusted *P* = 0.670; Fig 2E) and ρ = −0.16 for alpha-FT wPLI (95% CI −0.40 to 0.10; FDR-adjusted *P* = 0.670; Fig 2F). The five partial correlation estimates and their bootstrap 95% confidence intervals are summarized in Fig 2G. Thus, the adjusted analyses did not support a robust participant-level association between changes in positive affect and changes in the prespecified EEG-derived outcomes.

### Exploratory equivalence-oriented analysis of simulated sound

Exploratory equivalence analyses compared the sham + simulated sound group with the sham group. Complete results, including differences in change scores, 90% confidence intervals, outcome specific SESOI bounds, TOST results, and exploratory robustness analyses, are presented in Table 5. For positive affect, the 90% confidence interval for the difference between groups was not fully contained within the corresponding operationally defined SESOI bounds and excluded 0; therefore, equivalence was not established (Table 5; Fig 3J). For negative affect, comfort, satisfaction, and all five EEG outcomes, the 90% confidence intervals were also not fully contained within the corresponding equivalence margins, and equivalence was therefore not established for these outcomes. The estimates for comfort and satisfaction shown in Fig 3J similarly illustrate that their confidence intervals extended beyond the corresponding equivalence margins. Overall, equivalence between sham + simulated sound and sham manipulation was not established for any of the examined subjective or EEG outcomes. Taken together with the primary between group comparisons, these findings do not support the conclusion that adding a simulated cracking sound to sham manipulation reproduced the overall pattern of subjective and EEG responses observed after CRM. Because the equivalence margins were derived from observed pooled standard deviations rather than externally validated clinical thresholds, these analyses were considered exploratory and should not be interpreted as establishing clinical equivalence.

**Table 5:** Exploratory equivalence-oriented analysis comparing sham + simulated sound with sham manipulation.

Outcome	Difference(90%CI)	SESOI bounds	TOST *P*	Interpretation	Bootstrap(95%CI)	Permutation *P*	Leave-one-out	
**LOO estimate range**	**Same direction**
**positive affect**	−2.26(−3.94, − 0.57)	(−0.60, 0.60)	0.948	Equivalence not established	−2.26(−4.07, − 0.35)	0.030	(−2.58, − 1.87)	Yes
**negative affect**	0.15(−0.70,0.99)	(−0.30, 0.30)	0.381	Equivalence not established	0.15(−0.80,1.10)	0.773	(−0.06,0.36)	No
**comfort**	0.04(−1.39,1.47)	(−0.51, 0.51)	0.293	Equivalence not established	0.04(−1.67,1.67)	0.957	(−0.25,0.34)	No
**satisfaction**	−3.76(−7.74,0.21)	(−1.41, 1.41)	0.838	Equivalence not established	−3.76(−8.11,0.83)	0.111	(−4.76, − 3.09)	Yes
**Frontal alpha power**	−0.37(−0.74,0.01)	(−0.13, 0.13)	0.854	Equivalence not established	−0.37(−0.79,0.05)	0.108	(−0.45, − 0.30)	Yes
**Frontal alpha/beta**	−0.35(−0.81,0.11)	(−0.16, 0.16)	0.753	Equivalence not established	−0.35(−0.86,0.14)	0.210	(−0.43, − 0.27)	Yes
**Occipital IAF**	−0.03(−0.12,0.06)	(−0.03, 0.03)	0.489	Equivalence not established	−0.03(−0.13,0.07)	0.567	(−0.05, − 0.02)	Yes
**alpha-FP wPLI**	0.03(−0.01,0.06)	(−0.01, 0.01)	0.854	Equivalence not established	0.03(−0.01,0.06)	0.093	(0.02,0.03)	Yes
**alpha-FT wPLI**	0.01(−0.02,0.03)	(−0.01,0.01)	0.386	Equivalence not established	0.01(−0.03,0.03)	0.797	(0.01,0.02)	Yes

Between-group differences were calculated as Δ(sham + simulated sound) − Δsham, where Δ = P2 − P1; therefore, negative estimates indicate a smaller change in the sham + simulated sound group than in the sham group. Because established minimally important differences were unavailable, the outcome-specific SESOI bounds were operationally defined as ±0.20 × SD_pooled,Δ_, where SD_pooled,Δ_ was the pooled standard deviation of the change scores in the sham + simulated sound and sham groups. These bounds correspond to Cohen’s *d* = ±0.20. Equivalence was concluded only when the 90% confidence interval for the between-group difference fell entirely within the corresponding SESOI bounds. Because the margins were derived from the observed pooled standard deviations rather than externally validated clinical thresholds, the analysis was considered exploratory and should not be interpreted as establishing clinical equivalence.

CI, confidence interval; SESOI, smallest effect size of interest; TOST, two one-sided tests; LOO, leave-one-out; IAF, individual alpha frequency; wPLI, weighted phase lag index.

*Significant effect (*P* < 0.05).

## Discussion

### Principal findings and interpretation

This single-session, three-arm randomized controlled EEG study compared short-term subjective and EEG responses to the complete CRM procedure with those following sham manipulation with or without an externally generated simulated cracking sound [26]. CRM produced greater improvements in positive affect, comfort, and satisfaction than either control condition, whereas negative affect remained generally stable across groups. These subjective responses were accompanied by group differences in selected EEG-derived outcomes, particularly frontal alpha/beta ratio and alpha-band frontoparietal and frontotemporal functional connectivity. In contrast, the sham + simulated sound condition did not reproduce the overall pattern of subjective and EEG responses observed after the complete CRM procedure [27]. Furthermore, the adjusted brain–behavior analyses did not support a robust participant-level association between changes in positive affect and changes in the prespecified EEG-derived outcomes.

However, CRM differed from the control conditions in several interrelated respects, including active soft tissue release, positioning near the restrictive barrier, traction, HVLA thrust, joint movement, tactile and proprioceptive stimulation, possible vestibular input, and naturally occurring cavitation [9]. The observed between-group differences therefore cannot be attributed specifically to somatosensory input, the HVLA thrust, or any other individual component [28]. Similarly, the absence of a detectable additional response in the sham + simulated sound group does not exclude a contribution from naturally occurring cavitation or an interaction between auditory and mechanical components during CRM. Accordingly, the findings should be interpreted as responses to the complete CRM procedure rather than as evidence for a specific causal mechanism [6].

Notably, between-group EEG differences were detectable during the assessment window approximately 4–7 min after the intervention. This finding indicates that differences in the frontal alpha/beta ratio and alpha-band connectivity were measurable during this short-term post-intervention period. The delayed assessment therefore provides information about the EEG response several minutes after the procedure and should not be viewed solely as a methodological limitation. However, because EEG was not recorded during or immediately after the intervention, the present data cannot determine the onset, peak, or duration of these changes.

### EEG findings and exploratory brain-behavior associations

The EEG findings demonstrate neurophysiological differences that occurred alongside the subjective responses [29]. Among the spectral measures, frontal alpha power showed a group effect, whereas the frontal alpha/beta ratio demonstrated a clearer CRM-related pattern. Because increased alpha activity during eyes-closed rest has often been associated with lower arousal and relaxed internal attention, whereas beta activity is more closely related to cortical activation or alertness, the alpha/beta ratio may provide complementary information about relative spectral activity beyond alpha power alone [30]. Although the unadjusted pooled analysis initially suggested an association between frontal alpha/beta changes and positive-affect changes, this association was not retained after accounting for intervention group and participant sex. None of the five adjusted partial Spearman correlations was statistically significant, and all confidence intervals included zero. This finding indicates that the apparent pooled associations were likely influenced by between-group separation rather than reflecting a consistent participant-level brain–behavior relationship. Accordingly, the EEG and positive-affect findings should be interpreted as parallel group-level responses to CRM rather than as evidence of direct neural–affective coupling or mediation.

In contrast, occipital IAF did not differ significantly among the three groups. This finding is informative because IAF reflects the peak frequency of the individual alpha rhythm and may be relatively stable over short time intervals. The lack of a significant group difference in occipital IAF provides no evidence of a differential shift in individual alpha rhythm frequency among the three intervention conditions during the assessment window. Therefore, the observed EEG group differences were more evident in frontal spectral measures and alpha band functional connectivity than in posterior alpha peak frequency [31]. The alpha-band wPLI findings further suggest that CRM may influence interregional functional coupling. The CRM group showed greater changes in alpha-band fronto-parietal and fronto-temporal wPLI than the control groups, indicating that CRM may modulate not only local frontal oscillatory activity but also coordinated activity between frontal and posterior or temporal regions. These networks may be involved in attention, bodily awareness, and affective processing. Given the modest sample size, the connectivity findings should be interpreted as exploratory and do not establish a specific relaxation mechanism [32].

### Multisensory mechanisms and the contextual role of cracking sound

The present findings help distinguish an externally generated cracking-sound cue from the broader CRM procedure [33]. Adding simulated cracking sound to sham manipulation did not reproduce the overall pattern of subjective and EEG responses observed following complete CRM. However, this finding does not establish that naturally occurring cavitation is unimportant. Naturally occurring cavitation is temporally coupled to the thrust and may be accompanied by bone-conducted auditory, vibratory, tactile, and proprioceptive signals. It may therefore differ from an externally generated sound in timing, bodily localization, acoustic characteristics, and sensory congruence. Because naturally occurring cavitation was not prospectively recorded, its contribution could not be separated from the other components of CRM.

CRM is a multicomponent procedure involving positioning near the restrictive barrier, traction, rapid small-amplitude thrust, joint movement, and deformation of periarticular, muscular, fascial, and cutaneous tissues [34,35]. One possible hypothesis is that these mechanical events could engage cutaneous, muscular, joint, and deep cervical proprioceptive pathways and interact with visual, vestibular, and higher-order appraisal processes [36–38]. However, afferent activity was not measured in the present study, and these mechanisms therefore remain hypothetical. The observed responses should consequently be interpreted as responses to the complete CRM procedure rather than as evidence for any single sensory or mechanical mechanism.

The externally generated simulated sound may primarily have provided an auditory and contextual cue related to expectancy and treatment belief, whereas complete CRM combined auditory, tactile, mechanical, proprioceptive, and potentially vestibular inputs. The present findings therefore do not exclude a psychological or contextual contribution of cracking sound; rather, they indicate that the simulated auditory cue used under the present experimental conditions was insufficient to reproduce the broader response associated with complete CRM. These findings also have implications for clinical communication and future research. Cracking sound should not be presented as a necessary indicator of successful manipulation. Future mechanistic studies should independently manipulate auditory cues, tactile contact, passive cervical movement, end-range positioning, thrust-related input, vestibular contribution, and expectancy [39,40]. Combining EEG with autonomic measures such as heart rate variability, skin conductance, and respiratory indices may further clarify the physiological processes accompanying the short-term response to CRM.

### Limitations

This study has several limitations. First, participants were healthy volunteers; therefore, the findings should be interpreted as short term neurophysiological and experiential responses rather than as evidence of clinical efficacy. No dedicated baseline screening for dizziness, vertigo, or vestibular dysfunction was performed, so unreported or subclinical vestibular symptoms cannot be completely excluded. Second, post intervention EEG was recorded approximately 4–7 min after CRM, and transient responses occurring during or immediately after the intervention may therefore have been missed. In addition, the EEG cap was removed after P1 and reapplied before P2. Although electrode contact was standardized using the EmotivPRO contact quality indicator, numerical impedance values were unavailable, and equivalent electrode positioning and recording scale between sessions could not be confirmed. The finding for frontal absolute alpha power should therefore be considered preliminary. Third, the therapist could not be blinded, and participants could perceive whether a thrust had occurred. Treatment belief was assessed using a single nonvalidated question, and the simulated sound was not formally validated for perceived realism or similarity to naturally occurring cavitation. Expectancy and intervention awareness may therefore have influenced the outcomes reported by participants. The measures of comfort and satisfaction with the procedure were also exploratory and unvalidated, and the EEG metrics should not be regarded as specific markers of relaxation. Although sex distribution was comparable among groups and sex was included in exploratory sensitivity analyses, the study was not designed to evaluate sex specific intervention effects [41]. The modest sample size also limited the precision of analyses involving multiple EEG outcomes and exploratory brain behavior associations. Fourth, the equivalence margins were defined as ±0.20 times the observed pooled SD of the change scores because established minimally important differences were unavailable. These analyses should therefore be regarded as exploratory statistical assessments rather than as evidence of clinical equivalence. Finally, active soft tissue treatment was included only in the CRM group, and the respective contributions of soft tissue treatment, end range positioning, traction, thrust, tactile and proprioceptive stimulation, possible vestibular input, expectancy, and naturally occurring cavitation could not be separated. Naturally occurring cavitation was not systematically documented, and the simulated sound was not quantitatively matched to it in perceived location or realism. The findings should therefore be interpreted as responses to the complete CRM procedure rather than as evidence for any specific causal component.

## Conclusion

In this single session randomized controlled EEG study involving healthy volunteers, the complete CRM procedure produced greater short term improvements in positive affect, comfort, and satisfaction than sham manipulation with or without an externally generated simulated cracking sound. These subjective responses were accompanied by group differences in selected resting state EEG outcomes, particularly the frontal alpha/beta ratio and alpha band frontoparietal and frontotemporal functional connectivity. Adding a simulated cracking sound to sham manipulation did not reproduce the overall pattern of responses observed following the complete CRM procedure. However, because soft tissue release, end range positioning, traction, HVLA thrust, tactile and proprioceptive stimulation, possible vestibular input, and naturally occurring cavitation were not independently controlled, their respective contributions could not be determined. The findings therefore reflect responses to the complete CRM procedure and require confirmation in larger, independently recruited samples.

## Supporting information

S1 File CONSORT checklist. (DOCX)

S2 File Adapted I-PANAS-SF. (DOCX)

S3 File Comfort and satisfaction scale. (DOCX)

S4 File EEG Key Parameters and QC Thresholds. (DOCX)

S5 File Preprocessing Pipeline. (DOCX)

S6 File Clinical Trial Protocol. (DOCX)

S7 File Participant-level source data and data dictionary. (XLSX)

S8 File Exploratory sensitivity analyses. (XLSX)

S9 File Detailed descriptive statistics. (XLSX)

S10 File Detailed pairwise comparisons. (XLSX)

S11 File Statistical analysis and reproducibility code. (TXT)
