# Supplementary material for: Short-term subjective and EEG responses following cervical rotatory manipulation are not reproduced by simulated cracking sound: A randomized controlled study
Source: PLoS One. 2026 Oct 14;21(10):e0352526. doi: 10.1371/journal.pone.0352526 (PMC13667768; doi:10.1371/journal.pone.0352526)
Supplement: Supplementary file 1 [file pone.0352526.s001.docx]

**CONSORT checklist**

| **Section/topic** | **No** | **CONSORT 2025 checklist item description** | **Page** |
| --- | --- | --- | --- |
| **Title and abstract** | | | |
| **Title and structured abstract** | 1a | ☑Identification as a randomized trial | 1 |
|  | 1b | ☑Structured summary of the trial design, methods, results, and conclusions | 2-3 |
| **Open science** | | | |
| **Trial registration** | 2 | ☑Name of trial registry, identifying number (with URL) and date of registration | 3,6; S6 File |
| **Protocol and statistical analysis plan** | 3 | ☑Where the trial protocol and statistical analysis plan can be accessed | 30; S6 File |
| **Data sharing** | 4 | ☑Where and how the individual de-identified participant data (including data dictionary), statistical code and any other materials can be accessed | 30-31; S7/S11; OSF |
| **Funding and conflicts of interest** | 5a | ☑Sources of funding and other support (eg, supply of drugs), and role of funders in the design, conduct, analysis and reporting of the trial | 31 |
|  | 5b | ☑Financial and other conflicts of interest of the manuscript authors | 28 |
| **Introduction** | | | |
| **Background and rationale** | 6 | ☑Scientific background and rationale | 3-5 |
| **Objectives** | 7 | ☑Specific objectives related to benefits and harms | 5-7 |
| **Methods** | | | |
| **Patient and public involvement** | 8 | □Details of patient or public involvement in the design, conduct and reporting of the trial | no patient/public involvement |
| **Trial design** | 9 | ☑Description of trial design including type of trial (eg, parallel group or crossover), allocation ratio, and framework (for example, superiority, equivalence, non-inferiority or exploratory) | 6; S6 File |
| **Changes to trial protocol** | 10 | □Important changes to the trial after it commenced including any outcomes or analyses that were not prespecified, with reason | N/A |
| **Trial setting** | 11 | ☑Settings (such as community or hospital) and locations (eg, countries or sites) where the trial was conducted | 6 |
| **Eligibility criteria** | 12a | ☑Eligibility criteria for participants | 6-7; S6 File |
|  | 12b | ☑If applicable, eligibility criteria for sites and for individuals delivering the interventions (eg, surgeons or physiotherapists) | 8; S6 File |
| **Intervention and comparator** | 13 | ☑Intervention and comparator with sufficient details to allow replication. If relevant, where additional materials describing the intervention and comparator (eg, intervention manual) can be accessed | 8-10; S6 File |
| **Outcomes** | 14 | ☑Prespecified primary and secondary outcomes, including the specific measurement variable (eg, systolic blood pressure), analysis metric (for example, change from baseline, final value, time to event), method of aggregation (eg, median,  proportion), and time point for each outcome | 11-13; S2-S4 and S6 File |
| **Harms** | 15 | ☑How harms were defined and assessed (eg, systematically or non-systematically) | 7; S6 File |
| **Sample size** | 16a | ☑How sample size was determined, including all assumptions supporting the sample size calculation | 13; S6 File |
|  | 16b | □Explanation of any interim analyses and stopping guidelines | no interim analysis or stopping rule for this single-session trial |
| **Randomization:** | | | |
| **Sequence generation** | 17a | ☑Who generated the random allocation sequence and the method used | 7; S6 File |
|  | 17b | ☑Type of randomization and details of any restriction (eg, stratification, blocking and block size) | 7; S6 File |
| **Allocation concealment mechanism** | 18 | ☑Mechanism used to implement the random allocation sequence (eg, central computer/telephone; sequentially numbered, opaque, sealed containers), describing any steps to conceal the sequence until interventions were assigned | 7; S6 File |
| **Implementation** | 19 | ☑Whether the personnel who enrolled and those who assigned participants to the interventions had access to the random allocation sequence | 7; S6 File |
| **Blinding** | 20a | ☑Who was blinded after assignment to interventions (eg, participants, care providers, outcome assessors, data analysts) | 7-8; S6 File |
|  | 20b | ☑If blinded, how blinding was achieved and description of the similarity of interventions | 7-10; S6 File |
| **Statistical methods** | 21a | ☑Statistical methods used to compare groups for primary and secondary outcomes, including harms | 7, 13-15; S6 File |
|  | 21b | ☑Definition of who is included in each analysis (eg, all randomized participants), and in which group | 15 |
|  | 21c | ☑How missing data were handled in the analysis | 15 |
|  | 21d | ☑Methods for any additional analyses (eg. subgroup and sensitivity analyses), distinguishing prespecified from post hoc | 13-16, 19-22; S6, S8, S11 File |
| **Results** | | | |
| **Participant flow, including flow diagram** | 22a | ☑For each group, the numbers of participants who were randomly assigned, received intended intervention, and were analyzed for the primary outcome | 6, 15 |
|  | 22b | ☑For each group, losses and exclusions after randomization, together with reasons | 6, 15 |
| **Recruitment** | 23a | ☑Dates defining the periods of recruitment and follow-up for outcomes of benefits and harms | 6, 15 |
|  | 23b | □If relevant, why the trial ended or was stopped | No trial was not stopped early |
| **Intervention and**  **comparator delivery** | 24a | ☑Intervention and comparator as they were actually administered (eg, where appropriate, who delivered the intervention/comparator, how participants adhered, whether they were delivered as intended (fidelity)) | 8-10 |
|  | 24b | □Concomitant care received during the trial for each group | not reported: single-session intervention with no concomitant care |
| **Baseline data** | 25 | ☑A table showing baseline demographic and clinical characteristics for each group | 15-16 |
| **Numbers analyzed,**  **outcomes and estimation** | 26 | ☑For each primary and secondary outcome, by group:  • the number of participants included in the analysis  • the number of participants with available data at the outcome time point  • result for each group, and the estimated effect size and its precision (such as 95% confidence interval)  • for binary outcomes, presentation of both absolute and relative effect size | 15-20; S9, S10 File |
| **Harms** | 27 | ☑All harms or unintended events in each group | 15 |
| **Ancillary analyses** | 28 | ☑Any other analyses performed, including subgroup and sensitivity analyses, distinguishing pre-specified from post hoc | 16, 19-22; S6, S8, S11 File |
| **Discussion** | | | |
| **Interpretation** | 29 | ☑Interpretation consistent with results, balancing benefits and harms, and considering other relevant evidence | 22-29 |
| **Limitations** | 30 | ☑Trial limitations, addressing sources of potential bias, imprecision, generalisability, and, if relevant, multiplicity of analyses | 28, 29 |
