# Supplementary material for: Short-term subjective and EEG responses following cervical rotatory manipulation are not reproduced by simulated cracking sound: A randomized controlled study
Source: PLoS One. 2026 Oct 14;21(10):e0352526. doi: 10.1371/journal.pone.0352526 (PMC13667768; doi:10.1371/journal.pone.0352526)
Supplement: Supplementary file 2 [file pone.0352526.s002.docx]

**Adapted International Positive and Negative Affect Schedule-Short Form (Adapted I-PANAS-SF)**

**Instrument basis and assessment time frame**

The International Positive and Negative Affect Schedule-Short Form (I-PANAS-SF) contains five positive-affect items and five negative-affect items. The item set and domain assignments used in this study followed Thompson’s I-PANAS-SF [1]. Participants rated how strongly they experienced each affective state ‘right now’ immediately before and after the assigned intervention. The instrument was administered in Chinese. The Chinese item wording was based on the translated I-PANAS-SF examined by Liu et al. [2], while the present study retained all 10 items from Thompson’s original I-PANAS-SF, including “Alert.”

**Digital administration and continuous response format**

The questionnaire was administered through a mini-program using a continuous horizontal slider spanning 1 (lowest intensity) to 10 (highest intensity). The slider was not restricted to 10 fixed integer categories. Participants could stop the slider at intermediate positions, and the mini-program stored the selected position as a numeric value; therefore, individual item scores and summed domain scores could contain decimal values. The recorded continuous values were retained for analysis without rounding, binning, or conversion to the standard five category response format.

**Instruction to participants**

Please read each word and indicate the extent to which you feel this way right now. Move the slider to the position between 1 (lowest intensity) and 10 (highest intensity) that best represents your current feeling, and provide one response for each item.

**Table S2.1. Response-interface and data-recording specification**

| **Feature** | **Specification used in the present study** |
| --- | --- |
| Input control | Continuous horizontal slider |
| Displayed range | 1 (lowest intensity) to 10 (highest intensity) |
| Selectable positions | Continuous positions across the slider; not restricted to integer categories |
| Recorded item value | Numeric value from 1 to 10; decimal values permitted |
| Analysis value | Original recorded value |

**Table S2.2. Items and scoring**

| **Item no.** | **Dimension** | **Affective item(English)** | **情感项目（中文）** | **Score** |
| --- | --- | --- | --- | --- |
| 1 | Positive affect | Alert | 警觉性高的 | 1-10 |
| 2 | Positive affect | Inspired | 备受鼓舞的 | 1-10 |
| 3 | Positive affect | Determined | 意志坚定的 | 1-10 |
| 4 | Positive affect | Attentive | 注意力集中的 | 1-10 |
| 5 | Positive affect | Active | 有活力的 | 1-10 |
| 6 | Negative affect | Afraid | 害怕的 | 1-10 |
| 7 | Negative affect | Nervous | 紧张的 | 1-10 |
| 8 | Negative affect | Upset | 心烦的 | 1-10 |
| 9 | Negative affect | Hostile | 敌意的 | 1-10 |
| 10 | Negative affect | Ashamed | 害羞的 | 1-10 |

**Calculation of domain and change scores**

Positive affect was calculated as the sum of Alert, Inspired, Determined, Attentive, and Active. Negative affect was calculated as the sum of Afraid, Nervous, Upset, Hostile, and Ashamed. With five continuous item scores ranging from 1 to 10, each domain had a theoretical range of 5 to 50 and could take decimal values. Higher scores indicated greater positive or negative affect, respectively. For pre-post analyses, change scores were calculated as the post-intervention score minus the pre-intervention score on the continuous 5 - 50 metric. All participants completed all 10 items at both assessment time points. A domain score required responses to all five corresponding items; no item-level substitution, prorating, or imputation was performed.

**Evidence Base and Study-Specific Reliability**

The selection of the 10 items and their allocation to the positive- and negative-affect domains were based on Thompson’s development and validation of the I-PANAS-SF [1]. Subsequent Chinese-language psychometric research examined the translated instrument but ultimately supported a 9-item version after identifying “Alert” as a potentially problematic item among Chinese adolescents in Hong Kong [2]. This study therefore provides relevant evidence concerning the translated instrument and its two-domain structure but does not directly validate the 10-item version used in the present adult sample. Verhallen et al. administered an adapted PANAS using a 1 - 10 response format and analyzed the scores according to the corresponding range [3]. Their study provides a methodological precedent for using an expanded 1 - 10 PANAS response scale but does not constitute independent psychometric validation of the specific 10-item continuous-slider adaptation used in the present study.

As study-specific evidence of scoring integrity, the same slider interface, item set, scoring algorithm, and theoretical ranges were applied to all participants, intervention groups, and assessment time points. In the study, the five-item positive-affect subscale demonstrated acceptable-to-good internal consistency at pre-intervention (Cronbach’s α = 0.76) and post-intervention (α = 0.83). The five-item negative-affect subscale similarly demonstrated good internal consistency at pre-intervention (α = 0.81) and post-intervention (α = 0.84).

**References**

1. Thompson ER. Development and validation of an internationally reliable short-form of the Positive and Negative Affect Schedule (PANAS). J Cross Cult Psychol. 2007;38(2):227–242. doi:10.1177/0022022106297301.

2. Liu JD, You RH, Liu H, Chung PK. Chinese version of the International Positive and Negative Affect Schedule Short Form: factor structure and measurement invariance. Health Qual Life Outcomes. 2020;18:285. doi:10.1186/s12955-020-01526-6.

3. Verhallen AM, Renken RJ, Marsman J-BC, ter Horst GJ. Romantic relationship breakup: an experimental model to study effects of stress on depression (-like) symptoms. PLoS ONE. 2019;14(5):e0217320. doi:10.1371/journal.pone.0217320.
