# Supplementary material for: Short-term subjective and EEG responses following cervical rotatory manipulation are not reproduced by simulated cracking sound: A randomized controlled study
Source: PLoS One. 2026 Oct 14;21(10):e0352526. doi: 10.1371/journal.pone.0352526 (PMC13667768; doi:10.1371/journal.pone.0352526)
Supplement: Supplementary file 3 [file pone.0352526.s003.docx]

**Investigator developed comfort and procedure-related satisfaction scales**

**Table S3.1. Investigator developed State Comfort Scale**

**Instruction to participants:**

Please indicate how strongly you agree with each statement according to how you feel at this moment.

**Scoring:**

0 = Strongly disagree

1 = Disagree

2 = Neither agree nor disagree

3 = Agree

4 = Strongly agree

Total score: 0-32 points.The total score is calculated by summing the scores of the eight items. Higher scores indicate greater current comfort.

| **Domain** | **Item** | **Score** |
| --- | --- | --- |
| **Physical comfort** | 1. I feel physically comfortable at this moment. | 0–4 |
| **Neck–shoulder relaxation** | 2. My neck and shoulder region feels relaxed at this moment. | 0–4 |
| **Muscle tension** | 3. I feel little tension in my neck and shoulder region at this moment. | 0–4 |
| **Mental relaxation** | 4. I feel mentally relaxed at this moment. | 0–4 |
| **Calmness** | 5. I feel calm and at ease at this moment. | 0–4 |
| **Postural comfort** | 6. My current body position feels comfortable. | 0–4 |
| **Safety and ease** | 7. I feel safe and at ease during the current experimental procedure. | 0–4 |
| **Equipment/environment comfort** | 8. The current equipment and testing environment do not make me feel uncomfortable. | 0–4 |

The 8 item State Comfort Scale was developed by the research team for this single-session experiment. The items were selected to assess the participant’s current physical comfort, neck shoulder ease, muscle tension, mental ease, calmness, postural comfort, perceived safety, and equipment- or environment-related comfort. Each item was scored from 0 (“strongly disagree”) to 4 (“strongly agree”), yielding a total score ranging from 0 to 32. Higher scores indicated greater overall comfort and ease at the time of assessment. The same instructions and items were administered at P1 and P2, and the change score was calculated as P2 - P1.This investigator-developed scale has not undergone formal psychometric validation. Its construct validity, test retest reliability, and minimally important difference have not been established.

**Table S3.2. Investigator developed Procedure Related Satisfaction Scale**

**Instruction to participants:**

Please indicate your level of satisfaction with the current experimental experience. At P1, answer based on the procedure you have experienced so far. At P2, answer based on the procedure you have experienced up to this time point, including the intervention.

**Scoring:**

0 = Not at all satisfied

1–3 = Slightly satisfied

4–6 = Moderately satisfied

7–9 = Very satisfied

10 = Completely satisfied

Total score: 0-100 points. The total score is calculated by summing the scores of the ten items. Higher scores indicate greater procedure-related satisfaction.

| **Domain** | **Item** | **Score** |
| --- | --- | --- |
| **Overall satisfaction** | 1. Overall, I am satisfied with the experimental procedure I have experienced so far. | 0–10 |
| **Communication** | 2. I am satisfied with the explanation and communication provided by the researcher or operator. | 0–10 |
| **Professional manner** | 3. I am satisfied with the professional manner of the operator. | 0–10 |
| **Safety** | 4. I am satisfied with the safety of the procedure I have experienced so far. | 0–10 |
| **Acceptability** | 5. I find the current experimental procedure acceptable. | 0–10 |
| **Environment** | 6. I am satisfied with the testing environment, including quietness, lighting, and room temperature. | 0–10 |
| **Procedure arrangement** | 7. I am satisfied with the current arrangement and flow of the experimental procedure. | 0–10 |
| **Bodily experience** | 8. I am satisfied with my current bodily feeling. | 0–10 |
| **Relaxation experience** | 9. I am satisfied with my current level of relaxation. | 0–10 |
| **Willingness to continue/repeat** | 10. Under the same safety conditions, I would be willing to continue or receive a similar procedure again. | 0–10 |

The 10 item Procedure Related Satisfaction Scale was developed by the research team for this experiment. It assessed satisfaction with the experimental experience completed up to the time of assessment, including communication, operator manner, perceived safety, acceptability, testing environment, procedural arrangement, current bodily experience, perceived relaxation, and willingness to continue or repeat a similar procedure. Each item was scored from 0 (“not at all satisfied”) to 10 (“completely satisfied”), yielding a total score ranging from 0 to 100. Higher scores indicated greater procedure-related satisfaction.

At P1, which was completed after participant preparation, acclimatization, and the baseline assessment but before the assigned intervention, participants rated the experimental procedures and bodily experience encountered up to that point. At P2, participants rated the same domains after completing the assigned intervention and post-intervention assessment. The P2 - P1 score therefore represented the change in procedure-related satisfaction after the intervention was added to the experimental experience; it should not be interpreted as a pure measure of treatment satisfaction or relaxation.

This investigator-developed scale has not undergone formal psychometric validation. Its construct validity, test retest reliability, and minimally important difference have not been established.
