# Supplementary material for: Short-term subjective and EEG responses following cervical rotatory manipulation are not reproduced by simulated cracking sound: A randomized controlled study
Source: PLoS One. 2026 Oct 14;21(10):e0352526. doi: 10.1371/journal.pone.0352526 (PMC13667768; doi:10.1371/journal.pone.0352526)
Supplement: Supplementary file 4 [file pone.0352526.s004.docx]

**EEG Key Parameters & QC Thresholds**

**S4.1 Acquisition and preprocessing parameters**

| **Module** | **Parameter/Threshold** | **Value** |
| --- | --- | --- |
| **Acquisition system** | Hardware | 32-channel EMOTIV EPOC Flex (Emotiv, California, USA) |
| **Acquisition software** | Contact monitoring | Emotiv PRO contact-quality map |
| **Sampling** | Sampling rate | 128 Hz |
| **Montage** | Electrode locations | International 10-20 system |
| **Online reference** | Hardware configuration | Scalp channels recorded relative to CMS; DRL used for common-mode noise cancellation |
| **CMS/DRL location** | Placement | Bilateral ear sites |
| **Offline reference** | Final reference | Average reference after artifact removal and bad-channel interpolation |
| **Contact quality** | Recording criterion | All 32 recording channels required to display green ("good") status before P1 and P2 acquisition |
| **Numerical impedance** | Availability | Not provided by the device; quantitative P1-P2 impedance summaries were unavailable |
| **Cap reapplication** | Standardization | Same cap, electrode configuration, and anatomical positioning procedure for each participant; green status re-established before P2 |
| **Environment** | Room conditions | Dim lighting; temperature 24 ± 2 ℃; humidity 50 ± 5%; electromagnetic and acoustic disturbance minimized |
| **Software** | EEGLAB version | EEGLAB 13.0.0b |
| **Filtering** | Band-pass | 0.1-30 Hz |
| **Notch filter** | Application | No separate notch filter; the 30-Hz low-pass cutoff excluded 50-Hz mains activity |
| **Rest segment** | Buffer removal | 5 s at start& end |
| **Epoching** | Length / overlap | 2 s / 0% (non-overlapping); 256 samples per epoch |
| **Bad channel** | Flatline | ≥5 s |
|  | Noise | ＞median + 4 SD |
| **Bad channel policy** | Max bad channels | ≤3(interpolate allowed) |
| **Epoch rejection** | Amplitude | $\left\vert\mathrm{amp} \right\vert>1$00 μV |
|  | Peak-to-peak | p2p＞200 μV |
|  | Joint probability | ＞5 SD |
|  | Kurtosis | ＞5 SD |
| **Observation notes** | Use | Eye opening, swallowing, overt movement, and drowsiness used to guide visual confirmation; not automatic rejection markers |
| **ICA training copy** | High-pass | 1 Hz for ICA training only |
| **ICA algorithm** | Run settings | Infomax: runica, extended=1 |
| **ICA rejection** | Tool | ADJUST auto-label+manual |
|  | Back-projection | pop_subcomp |
| **Interpolation** | Method | Spherical interpolation of previously removed bad channels |
| **Minimum usable data** | Clean duration | ≥ 120 s |
| **Preprocessing blinding** | File coding | Participant and time-point codes used without group labels during preprocessing |
| **QC log** | Required fields | #bad chans, #interp, epoch reject %, #rejected ICs, clean duration |

**S4.2 Predefined EEG regions of interest**

| **ROI** | **Channels** | **Use** | **n** |
| --- | --- | --- | --- |
| **Frontal** | Fz, Fp1, Fp2, F3, F4, F7, F8 | Frontal alpha power; frontal alpha/beta ratio; source ROI for FP and FT wPLI | 7 |
| **Central** | Cz, FC1, FC2, C3, C4, FC5, FC6 | Anatomical organization and exploratory whole-scalp summaries; not one of the five prespecified endpoints | 7 |
| **Parietal** | Pz, CP1, CP2, P3, P4, CP5, CP6 | Target ROI for FP wPLI | 7 |
| **Occipital** | Oz, O1, O2 | Occipital individual alpha frequency | 3 |
| **Temporal** | T7, T8 | Target ROI for FT wPLI | 2 |

S4.3 Spectral and connectivity feature extraction

| **Feature / step** | **Definition and implementation** |
| --- | --- |
| **Power spectral density** | Welch method applied to retained 2-s epochs using a Hamming window, no overlap, and a 256-point FFT (nominal frequency resolution 0.5 Hz); epoch spectra were averaged at channel level |
| **Frontal alpha power** | Mean alpha-band PSD density across 8-13 Hz, averaged across the seven frontal channels; reported on the absolute scale |
| **Frontal beta power** | Mean beta-band PSD density across 13-30 Hz, averaged across the seven frontal channels |
| **Frontal alpha/beta ratio** | Frontal alpha-band estimate divided by the corresponding frontal beta-band estimate at each time point |
| **Occipital IAF** | Frequency of the maximum PSD peak within 8-13 Hz after averaging the spectra across Oz, O1, and O2 |
| **Alpha-band analytic signal** | Preprocessed EEG filtered to 8-13 Hz; Hilbert transform used to derive analytic signals |
| **wPLI implementation** | For each channel pair and 2-s segment, wPLI was calculated from the imaginary component of the analytic-signal cross-product as $wPLI=\frac{\left\vert E\left[ Im(S_{xy}) \right] \right\vert}{E\left[ \left\vert Im(S_{xy}) \right\vert\right]}$ |
| **Segment averaging** | Channel-pair wPLI values were averaged across all retained 2-s segments to obtain one P1 and one P2 matrix per participant |
| **ROI-pair averaging** | Mean of all corresponding cross-ROI channel pairs: 49 pairs for FP and 14 pairs for FT |
| **Change scores** | For all prespecified EEG outcomes, delta = P2 - P1 |
| **Power transformation** | The primary frontal alpha-power analysis used the untransformed absolute-power scale; no relative-power normalization was applied |
| **Whole-scalp analyses** | Whole-matrix results were used for exploratory visualization only and were not included in the prespecified inferential endpoint family |

S4.4 Summary of retained artifact-free EEG data

| **Group** | **Time point** | **Recordings (n)** | **Retained epochs** | **Retained duration (s)** | **Epoch rejection (%)** |
| --- | --- | --- | --- | --- | --- |
| CRM | P1 | 18 | 76.94±2.88 | 153.89±5.76 | 9.48±3.39 |
| CRM | P2 | 18 | 77.11±3.25 | 154.22±6.50 | 9.28±3.83 |
| sham | P1 | 18 | 77.28±3.27 | 154.56±6.54 | 9.08±3.85 |
| sham | P2 | 18 | 77.33±2.63 | 154.67±5.27 | 9.02±3.10 |
| sham + simulated sound | P1 | 18 | 77.22±3.14 | 154.44±6.27 | 9.15±3.69 |
| sham + simulated sound | P2 | 18 | 77.17±2.81 | 154.33±5.63 | 9.22±3.31 |

Values are presented as mean ± SD. A maximum of 85 non-overlapping 2 s epochs was available from each recording. All 108 recordings retained at least 60 artifact-free epochs (120 s), and no recording or participant was excluded on EEG quality-control grounds.
