# Supplementary material for: Short-term subjective and EEG responses following cervical rotatory manipulation are not reproduced by simulated cracking sound: A randomized controlled study
Source: PLoS One. 2026 Oct 14;21(10):e0352526. doi: 10.1371/journal.pone.0352526 (PMC13667768; doi:10.1371/journal.pone.0352526)
Supplement: Supplementary file 5 [file pone.0352526.s005.docx]

**Preprocessing Pipeline**

Acquisition prerequisite. Before P1 and P2, hair, conductive gel, and electrode contact were adjusted until all 32 recording channels displayed green ("good") status on the Emotiv PRO contact-quality map. After cap reapplication, the complete check was repeated before P2.

Blinding. Files were identified using participant and time-point codes without intervention-group labels during preprocessing. The same thresholds and decision rules were applied to P1 and P2 in all three groups.

| **No.** | **Step** | **Input** | **Operation** | **Output** |
| --- | --- | --- | --- | --- |
| 1 | Acquisition QC and coding | Participant; P1/P2 | Confirm 32 green channels; verify 128-Hz recording; assign participant/time-point filename without group label | Contact criterion; coded filename |
| 2 | Import & montage | Raw EEG | Import; load 10–20 channel locations; verify sampling rate & labels | Log: subject ID, timepoint (P1/P2), file name |
| 3 | Initial bad-channel screening | Continuous EEG | Flag channels with flatline ≧5 s or noise > channel median + 4 SD; temporarily remove flagged channels; maximum three | Bad-channel identities and count |
| 4 | Main-data filtering | Continuous EEG without flagged channels | Band-pass filter 0.1-30 Hz using pop_eegfilt/eegfilt. No separate notch filter. | Filtered main dataset; filter settings |
| 5 | Rest segment & epoching | Filtered data | Extract 3-min eyes-closed rest; remove first/last 5 s; epoch into 2 s non-overlapping segments | Log: total epochs |
| 6 | Epoching | 170 s analysis segment | Divide into 2 s non-overlapping epochs (256 samples per epoch; maximum 85 epochs before rejection) | Epoched dataset; total epoch count |
| 7 | Automated and visual epoch rejection | Epoched EEG | Reject epochs with \|amplitude\| >100 microV, peak-to-peak >200 microV, joint probability >5 SD, or kurtosis >5 SD. Use acquisition notes only to guide visual confirmation | Rejected epochs; rejection percentage; retained duration |
| 8 | ICA-copy training | Clean epochs | Duplicate dataset; high-pass 1 Hz on ICA-copy only; run runica (extended=1) | Output: ICA weights; log ICA settings |
| 9 | ADJUST + manual IC removal | ICA results | ADJUST auto-label; manual confirmation; remove ICs with pop_subcomp | Log: rejected IC indices & count |
| 10 | Channel restoration and reference | ICA-cleaned data | Spherically interpolate previously removed bad channels; restore montage; apply average reference | Complete channel set; final reference; interpolation log |
| 11 | Final QC & export | Clean data | Check waveform/spectrum; compute clean duration (≥120 s); export .set/.fdt + QC log | Output: cleaned data + QC log |
| 12 | Spectral feature extraction | Final clean epochs | Welch PSD with 2-s Hamming windows; derive frontal alpha power, frontal alpha/beta ratio, and occipital IAF using predefined ROIs | Participant-level spectral outcomes at P1/P2 |
| 13 | Connectivity feature extraction | Final clean epochs | Filter to 8-13 Hz; obtain analytic signals with the Hilbert transform; compute segment-level channel-pair wPLI; average across segments and predefined ROI pairs | P1/P2 FP and FT wPLI; whole-matrix visualization data |
| 14 | Export and analysis lock | Clean data and derived features | Export cleaned .set/.fdt files, QC record, and participant-level features; reveal group allocation only after preprocessing outputs are finalized | Analysis-ready EEG outcomes and QC documentation |
