# Supplementary material for: Short-term subjective and EEG responses following cervical rotatory manipulation are not reproduced by simulated cracking sound: A randomized controlled study
Source: PLoS One. 2026 Oct 14;21(10):e0352526. doi: 10.1371/journal.pone.0352526 (PMC13667768; doi:10.1371/journal.pone.0352526)
Supplement: Supplementary file 6 [file pone.0352526.s006.docx]

**Clinical Trial Protocol**

**Name of Research Project:** Short-term subjective and EEG responses following cervical rotatory manipulation are not reproduced by simulated cracking sound: a randomized controlled study

**1. Purpose of research**

Cervical rotatory manipulation (CRM) is commonly used in traditional Chinese orthopedic manipulation and is frequently accompanied by a cracking sound. Patients and therapists may regard this sound as a sign that manipulation has occurred. However, it remains unclear whether adding an externally generated simulated cracking sound to a sham manipulation can reproduce the short-term subjective and resting-state EEG responses observed following the complete CRM procedure.

The primary aim is to compare short-term subjective and electroencephalography (EEG) responses among CRM, sham manipulation, and sham manipulation plus simulated cracking sound in healthy volunteers. The study is designed to determine whether the simulated sound cue, when added to sham manipulation, reproduces the overall response pattern observed after the complete CRM procedure. The study is not designed to isolate the independent causal contributions of active soft-tissue release, end-range positioning, traction, HVLA thrust, joint movement, tactile or proprioceptive stimulation, possible vestibular input, naturally occurring cavitation, expectancy, or other contextual components. Accordingly, findings will be interpreted at the level of the complete CRM procedure rather than as evidence for a specific mechanism.

**2. Inclusion and exclusion criteria**

**2.1 Inclusion criteria**

Participants will be eligible if they meet all of the following criteria:

(a) age between 20 and 50 years;

(b) no cervical disc herniation or other cervical structural lesions requiring treatment, as confirmed by imaging and clinical screening;

(c) no history of tumor, major trauma, or cervical spine surgery;

(d) body mass index (BMI) < 26 kg/m²;

(e) Self-Rating Anxiety Scale (SAS) standard score < 53 and Self-Rating Depression Scale (SDS) standard score < 53;

(f) no massage, manipulation, physical therapy, or related interventions within the previous month;

(g) willingness to participate and ability to provide written informed consent.

**2.2 Exclusion criteria**

Participants will be excluded if they meet any of the following criteria:

(a) imaging evidence of cervical deformity or high-risk anatomical abnormalities, such as block vertebrae, os odontoideum, or basilar invagination;

(b) severe cardiovascular or cerebrovascular disease, coagulation disorders, or other conditions that may increase the risk of cervical manipulation;

(c) history of cranial or spinal trauma or surgery;

(d) autoimmune or infectious diseases;

(e) pregnancy or lactation;

(f) implanted electronic devices, metal implants, or other conditions that may affect EEG acquisition or intervention safety;

(g) marked fear of manipulation or inability to cooperate with eyes-closed resting-state EEG recording.

**2.3 Withdrawal criteria**

Participants may withdraw from the study at any time by revoking informed consent. Investigators may discontinue participation if a serious adverse event occurs, if newly developed comorbidities or complications make continued participation inappropriate, or if the participant fails to complete EEG acquisition.

**3. Specific implementation steps**

**3.1 Study design and registration**

This study is designed as a single-session, three-arm, parallel randomized controlled trial. Eligible participants will be randomly allocated in a 1:1:1 ratio to one of three groups: the CRM group, the sham manipulation group, or the sham manipulation plus simulated sound group.

The study protocol has been approved by the Ethics Committee of The Affiliated TCM Hospital of Guangzhou Medical University (Approval No.: 2025NK022; approval date: 7 March 2025). The trial has been registered with the International Traditional Medicine Clinical Trial Registry (Registration No.: ITMCTR2025001112; registration date: 23 March 2025). Written informed consent will be obtained from all participants before enrollment.

**3.2 Randomization, allocation concealment, and blinding**

The randomization sequence will be generated by an independent researcher using a computer-based random number generator with randomly permuted block sizes of 6 and 9 and a 1:1:1 allocation ratio. Allocation concealment will be ensured using sequentially numbered, opaque, sealed envelopes prepared by a staff member not involved in recruitment, intervention delivery, outcome assessment, EEG preprocessing, or statistical analysis.

After enrollment and completion of baseline assessments, the therapist will open the next envelope in numerical order to determine the participant’s group assignment. Outcome assessors, EEG preprocessing personnel, and statistical analysts will be blinded to group allocation. Participants will be blinded to the study hypothesis; however, full participant blinding to the presence or absence of an HVLA thrust is not feasible because the thrust is perceptible. At the end of the experiment, participants will be asked whether they believe they have received CRM, and their responses will be used for a descriptive assessment of blinding integrity.

**3.3 Intervention procedures**

All interventions will be delivered in a single session by a researcher with more than 5 years of clinical experience in CRM. Before the principal intervention procedure, all participants will receive approximately 15 s of therapist contact. In the CRM group, this will consist of active soft-tissue release as part of the complete clinical CRM procedure. In the two control groups, participants will receive light contact of similar duration without therapeutic soft-tissue techniques. The active soft-tissue component is not reproduced in the control groups because the study compares the complete CRM procedure with sham conditions rather than isolating the effect of the HVLA thrust. This difference is recognized as a limitation for component-specific causal interpretation.

**3.3.1 CRM group**

Participants will be seated, and the operator will stand behind them. Before the thrust procedure, a brief standardized soft-tissue release will be applied for approximately 15 s. This release will mainly consist of acupoint-based pressing, kneading, and plucking techniques. The operator will press and knead the paraspinal muscles around the cervical spinous processes and, when tender points or palpable cord-like taut bands are encountered, will gently pluck the surrounding soft tissue.

After soft-tissue release, participants will be instructed to flex the neck forward. The operator will place the thumb of one hand against one side of the C5-C6 level, with the remaining fingers on the contralateral side, while the other hand supports the mandible. Because participants are healthy volunteers without a clinically indicated symptomatic side, CRM will be performed bilaterally using the same standardized procedural sequence on both sides. For each side, slow upward traction will be applied and the head will be rotated approximately 45°. After the restrictive barrier is reached, the operator will briefly pause and deliver a controlled, rapid, small-amplitude thrust with an additional rotation of approximately 5°.

The procedure may be accompanied by naturally occurring cavitation or an audible cracking sound. No sound will be played through the headset in the CRM group, and no repeated thrust will be performed solely to elicit an audible sound. The occurrence, number, timing, and acoustic characteristics of naturally occurring cavitation are not prospectively recorded or standardized. Accordingly, the CRM condition represents the complete clinical procedure rather than an isolated HVLA thrust, and a valid subgroup comparison based on natural cavitation cannot be prespecified.

**3.3.2 Sham group**

The sham manipulation procedure will be designed to match the CRM condition as closely as possible in participant positioning, therapist contact, contact points, procedural duration, and standardized verbal instructions. Participants will be seated in the same position, and the operator will stand behind them. The same bone-conduction headset will be worn, but no sound will be played. Before passive neck positioning, a matched 15 s light manual contact procedure will be applied around the cervical paraspinal region. This procedure will involve gentle contact only, without deep pressing, kneading, plucking of taut bands, or therapeutic soft-tissue release.

The operator will then place the hands in positions similar to those used in the CRM group and gently position the participant’s neck within a comfortable range. The procedure will not approach the restrictive barrier, and no HVLA thrust will be delivered. Passive positioning and gentle stretching will be applied bilaterally within a comfortable range.

**3.3.3 Sham + simulated sound group**

The sham manipulation procedure will be identical to that used in the sham group, including the matched 15 s light manual contact procedure before passive neck positioning. During passive neck rotation, a brief standardized bone-conducted sound burst will be delivered through the Newman M110 headset to mimic a cracking sound. Each burst will last approximately 0.2 - 0.5 s and will be delivered 1 - 2 times per session, synchronized with the peak rotation moment. The waveform and playback intensity will be computer-controlled and kept constant across participants. Playback intensity will be standardized through pretrial technical calibration to ensure audibility and comfort. This calibration is not a formal validation of realism, intensity, anatomical location, or similarity to naturally occurring cavitation. No HVLA thrust will be delivered in this group.

**3.4 Assessment procedure and subjective outcomes**

Participants will first complete a 15-min acclimatization period with eyes closed. Baseline resting state EEG will then be recorded for 3 min and defined as P1. The EEG cap will be removed after P1 in all participants because the interventions require access to the occipital and cervical regions. After the assigned intervention, the cap will be reapplied using the same electrode configuration and anatomical positioning procedure. Reapplication is expected to require approximately 3 min. P2 EEG recording will begin approximately 4 min after completion of the intervention and continue for 3 min; the EEG assessment window will therefore represent approximately 4 - 7 min after the intervention.

Subjective outcomes will include the Adapted International Positive and Negative Affect Schedule Short Form (Adapted I-PANAS-SF), an eight-item State Comfort Scale, and a ten-item Procedure Related Satisfaction Scale developed by the investigators. The Adapted I-PANAS-SF will be administered in Chinese through a mini-program using a continuous slider ranging from 1 to 10. Five positive-affect items and five negative-affect items will be summed separately, yielding a theoretical range of 5 - 50 for each domain. Decimal values generated by the slider will be retained without post hoc rounding, recategorization, conversion, or rescoring. The State Comfort Scale will range from 0 to 32, and the Procedure Related Satisfaction Scale from 0 to 100, with higher values indicating greater comfort or satisfaction. The satisfaction change score will be interpreted as a change in cumulative procedure-related satisfaction rather than as a pure measure of treatment satisfaction or relaxation. Full instrument details are provided in S2 and S3 Files.

**3.5 Safety monitoring**

Safety will be monitored during the intervention and immediately after the intervention by proactive inquiry and direct observation. Recorded adverse events will include, but will not be limited to, neck pain or discomfort, dizziness, nausea, upper-limb numbness, transient neurological symptoms, and skin discomfort. For each adverse event, the onset time, duration, severity, management, outcome, and relatedness to the intervention will be documented. In the event of a serious adverse event, the participant will immediately discontinue the intervention and receive appropriate medical assessment and management. The event will be reported to the ethics committee according to institutional requirements.

**4. Judgment and evaluation methods of experimental efficacy**

**4.1 Subjective outcomes**

The prespecified primary subjective outcome is the P1-to-P2 change in positive affect assessed with the Adapted I-PANAS-SF. Secondary subjective outcomes include changes in negative affect, comfort, and procedure-related satisfaction. Subjective outcomes will be retained at the original P1 and P2 measurement levels for the primary mixed-effects analysis so that within-participant repeated observations and the group × time interaction can be modeled directly.

**4.2 EEG data acquisition and analysis**

EEG will be recorded using a 32-channel EMOTIV EPOC Flex system (Emotiv, California, USA) at a sampling rate of 128 Hz, with scalp electrode positions based on the international 10-20 system. Scalp channels will be recorded relative to the CMS electrode, with the DRL electrode used for common-mode noise cancellation; CMS and DRL will be positioned at the bilateral ear sites. After artifact removal and interpolation of bad channels, recordings will be re-referenced to the average reference.

Before both P1 and P2 recordings, hair, conductive gel, and electrode contact will be adjusted until all 32 recording channels display green (“good”) status on the EmotivPRO contact-quality map. The device provides a categorical contact-quality indicator rather than numerical impedance values. Following cap reapplication, the same cap, electrode configuration, and anatomical positioning procedure will be used, and the complete contact-quality check will be repeated before P2. This standardization cannot guarantee identical electrode position, electrode-scalp contact, impedance, or absolute signal scaling between sessions; this limitation is especially relevant to interpretation of absolute spectral power.

EEG preprocessing will be performed in MATLAB 2013b using EEGLAB 13.0.0b. Continuous data will be band-pass filtered from 0.1 to 30 Hz. No separate notch filter will be applied because the 30 Hz low-pass cutoff excludes 50-Hz mains activity. Channels with a flatline of at least 5 s or noise exceeding the channel median by more than 4 SD will be flagged as bad channels; no more than three channels may be interpolated. The first and last 5 s of each 3 min recording will be removed, and the remaining data will be divided into non-overlapping 2 s epochs.

Epochs will be rejected when absolute amplitude exceeds 100 µV, peak-to-peak amplitude exceeds 200 µV, or joint probability or kurtosis exceeds 5 SD. Observed eye opening, swallowing, overt body movement, and drowsiness will be recorded as qualitative aids for visual artifact review; these observations will not be treated as statistical covariates or automatic rejection events. Independent component analysis will be performed using extended Infomax on a 1-Hz high-pass-filtered copy of the data. ICA weights will be transferred to the corresponding 0.1-30 Hz dataset, and artifactual components will be identified using ADJUST with visual confirmation. Flagged bad channels will then be spherically interpolated, followed by average re-referencing. Recordings will be retained only when at least 120 s (60 epochs) of artifact-free EEG remain. Detailed thresholds and the sequential workflow are provided in S4 and S5 Files.

**4.3 Prespecified EEG-derived outcomes**

The five prespecified EEG-derived outcomes are frontal alpha power, frontal alpha/beta ratio, occipital individual alpha frequency (IAF), alpha-band frontoparietal weighted phase lag index (wPLI), and alpha-band frontotemporal wPLI. Predefined electrode compositions for frontal, parietal, temporal, and occipital regions are provided in S4 File.

Power spectral density will be estimated from retained epochs using Welch’s method with a 2 s Hamming window. Frontal alpha power will be calculated over 8-13 Hz and frontal beta power over 13-30 Hz; the frontal alpha/beta ratio will be calculated at each time point. The primary frontal alpha-power analysis will use the untransformed absolute power scale. Occipital IAF will be defined as the frequency of the maximum power spectral density peak within 8-13 Hz.

For connectivity analyses, preprocessed EEG will be filtered to 8-13 Hz and analytic signals will be obtained using the Hilbert transform. wPLI will be calculated for individual channel pairs, averaged across retained segments, and then averaged across predefined frontal-to-parietal and frontal-to-temporal region pairs. Whole-matrix connectivity will be used only for exploratory visualization. For between-group EEG analyses, participant-level change scores will be calculated as Δ = P2 - P1.

**5. Sample size estimation and statistical analysis**

**5.1 Sample size estimation**

An a priori sample size calculation was performed using G*Power version 3.1. The test family was specified as F tests and the statistical test as fixed effects omnibus one-way analysis of variance, corresponding to an omnibus comparison of P2 - P1 changes in positive affect among the three groups. Because G*Power does not directly implement the linear mixed effects model used for the final primary analysis, one-way analysis of variance of change scores was used as an approximation for sample size planning. Based on preliminary work, an omnibus standardized effect size of Cohen’s *f* = 0.55 was assumed. With three groups, α = 0.05, 90% power, and equal allocation, the minimum required sample size was 45 participants, or 15 per group. Recruitment could screen more volunteers than the minimum randomized sample to allow for ineligibility or refusal before randomization. The standardized omnibus effect, rather than a single expected pairwise mean difference, was the input used for the calculation.

**5.2 Core statistical analysis plan**

Analyses will be performed using GraphPad Prism and R. Continuous variables will be summarized as mean ± standard deviation and categorical variables as counts and percentages. Baseline characteristics will be compared among groups using one-way analysis of variance, Pearson’s chi-square test, or Fisher’s exact test as appropriate. Change scores will be defined as Δ = P2 - P1.

Subjective outcomes will be analyzed using linear mixed-effects models with group, time, and group × time interaction as fixed effects and participant ID as a random intercept. The group × time interaction will be the primary test of differential pre-to-post change among groups. Significant interactions will be followed by the planned between-group comparisons of change. If an interaction is not statistically significant, the full model will be retained rather than refitted as a reduced additive model, and main effects will not be interpreted as differential intervention effects.

Each of the five prespecified EEG-derived change scores will be analyzed using a separate one-way analysis of variance with intervention group as the between-participant factor. The planned contrasts will be expressed consistently as the first-listed group minus the second-listed group: sham - CRM, sham + simulated sound - CRM, and sham + simulated sound - sham. Pairwise EEG effect sizes will be reported as signed Cohen’s *d*, calculated as the between-group difference in mean change divided by the pooled standard deviation of the two groups’ change scores.

The Benjamini-Hochberg false discovery rate procedure will be applied across the five primary EEG omnibus group-effect tests. Model assumptions will be assessed using residual diagnostics; homogeneity of variance for the one-way analyses of variance will be evaluated using Levene’s test, and influential observations will be examined using standardized residuals and Cook’s distance where applicable. Missing outcome data, if any, will be reported explicitly.

**5.3 Exploratory equivalence-oriented analysis**

An exploratory equivalence-oriented analysis will compare sham + simulated sound with sham using the two one-sided tests procedure. Because established minimally important differences are unavailable for the subjective change scores and EEG-derived outcomes, a standardized smallest effect size of interest of Cohen’s *d* = ±0.20 will be used operationally as a small-effect threshold. For each outcome, this standardized criterion will be converted to the original units as ±0.20 × SD_pooled,Δ_, where SD_pooled_,Δ is the pooled standard deviation of the change scores in the sham + simulated sound and sham groups. Equivalence will be concluded only when the 90% confidence interval for the between-group difference lies entirely within the corresponding outcome-specific bounds.
